# The Description and Prediction of Incidence, Prevalence, Mortality, Disability-Adjusted Life Years Cases, and Corresponding Age-Standardized Rates for Global Diabetes

**DOI:** 10.1007/s44197-023-00138-9

**Published:** 2023-07-03

**Authors:** Jianran Sun, Wan Hu, Shandong Ye, Datong Deng, Mingwei Chen

**Affiliations:** 1 Department of Endocrinology, Division of Life Science and Medicine, The First Affiliated Hospital of USTC, University of Science and Technology of China, Hefei, 230001 Anhui China; 2 Department of Epidemiology and Biostatistics, School of Public Health, Anhui Medical University, 81 Meishan Road, Hefei, 230032 Anhui China; 3 Department of Endocrinology, The First Affiliated Hospital of Anhui Medical University, 218 Jixi Road, Hefei, 230022 Anhui China

**Keywords:** Global diabetes burden, Evaluation, Prediction

## Abstract

**Objective:**

Diabetes is a life-long disease that poses a serious threat to safety and health. We aimed to assess the disease burden attributable to diabetes globally and by different subgroups, and to predict future disease burden using statistical models.

**Methods:**

This study was divided into three stages. Firstly, we evaluated the disease burden attributable to diabetes globally and by different subgroups in 2019. Second, we assessed the trends from 1990 to 2019. We estimated the annual percentage change of disease burden by applying a linear regression model. Finally, the age-period-cohort model was used to predict the disease burden from 2020 to 2044. Sensitivity analysis was performed with time-series models.

**Results:**

In 2019, the number of incidence cases of diabetes globally was 22239396 (95% uncertainty interval (UI): 20599519–24058945). The number of prevalence cases was 459875371 (95% UI 423474244–497980624) the number of deaths cases was 1551170 (95% UI 1445555–1650675) and the number of disability-adjusted life years cases was 70880155 (95% UI 59707574–84174005). The disease burden was lower in females than males and increased with age. The disease burden associated with type 2 diabetes mellitus was greater than that with type 1; the burden also varied across different socio-demographic index regions and different countries. The global disease burden of diabetes increased significantly over the past 30 years and will continue to increase in the future.

**Conclusion:**

The disease burden of diabetes contributed significantly to the global disease burden. It is important to improve treatment and diagnosis to halt the growth in disease burden.

**Supplementary Information:**

The online version contains supplementary material available at 10.1007/s44197-023-00138-9.

## Introduction

Diabetes is a chronic metabolic disease characterized by hyperglycemia [1]. This is a life-long disease with complex pathogenesis, multiple risk factors, and a long course [2–4]. Diabetes is related to insulin resistance and insufficient insulin secretion [5] and poses a serious threat to safety, human life, and health [6]. Diabetes can cause damage to several organs, including the heart, brain, kidneys, and eyes. Furthermore, severe cases can lead to disability and even death [7–9]. According to the International Diabetes Federation (IDF), there were 463 million adults with diabetes worldwide in 2019, an average of 1 in 11 adults. Furthermore, there were 4.2 million individuals who died from diabetes and its complications, accounting for about 11.3% of all global deaths [10]. Another study reported that there were 67.9 million disability-adjusted life years (DALYs) attributable to diabetes globally in 2017. In the global ranking of DALYs caused by disease, DALYs caused by diabetes ranks ninth for females and eighth for males [11–13]. In addition, alongside changes in dietary structure and irregular living habits in modern society over recent years, the incidence, morbidity, and mortality of incidence has shown an increasing trend year by year [14]. According to the tenth edition of the Diabetes Map released by the IDF, there were 537 million patients in diabetes globally in 2021, accounting for 10.5% of the global population. Compared with data reported in the ninth edition of the Diabetes Map, the prevalence rate of diabetes increased by 12.9% overall. The number of diabetic patients is expected to reach 578 million by 2030, and estimated to reach 783 million worldwide by 2045 [15]. The control and treatment of this disease requires substantial medical resources [16]. Other studies have shown that DALYs increased by 30.0% from 2007 to 2017, years of life lost (YLL) increased by 29.9%, and years lived with disability (YLD) increased by 30.1% [11–13]. Moreover, the economic expenditure related to diabetes globally was substantial, reaching 1054 billion dollars by 2045 [17]. The widespread prevalence of diabetes has brought huge burden to the social and economic development of the world, and the situation is not optimistic. Diabetes has become a global public health problem. How we can effectively treat diabetes, slow down the occurrence of complications, and greatly improve the life quality of patients, have become critical medical problems that needed to be solved urgently.

Over recent years, numerous models have been used to assess and predict the disease burden associated with diabetes. The main model used to assess disease burden is the linear regression model. For example, the Global Burden of Disease (GBD) 2019 Dementia Prediction Collaborative Group [18] used a linear regression model to investigate changes in the global prevalence of dementia. Li [19] used a linear regression model to evaluate the disease burden associated with esophageal cancer. Many models have been used for disease burden; the most common models are the age-period-cohort (APC) model and the time series model. Ji [20] used the APC model to predict the incidence of hepatitis B in males and females of specific age groups. In another study, Akita [21] used the APC model to predict the mortality associated with hepatocellular carcinoma and recapitulated the observed mortality. Zheng [22] explored the feasibility of using the auto-regressive integrated moving average model (ARIMA) and the Elman neural network model to predict the incidence of hepatitis B. Ceylan [23] used the ARIMA model to predict the prevalence trend of COVID-19 in the three countries most affected by COVID-19 in Europe, including Spain, Italy, and France. In another study, Fu [24] used the exponential smoothing model (ES) model to predict the incidence of acute upper gastrointestinal bleeding. However, no previous study has applied these models to assess and predict the disease burden related to diabetes. This study aimed to complement and refine this aspect of analysis by applying these common and reliable models.

Accurate disease burden data is an important basis for scientific development and the timely adjustment of health policies and strategies. Such data can contribute to the development of clear prevention and control priorities, and help to evaluate the efficacy of measures. Some studies have examined the disease burden associated with diabetes in specific regions. These studies found that although the mortality rate associated with diabetes was declining, the burden of diabetes was still increasing [25]. Other studies showed that the impact of diabetes on cancer was increasing [26]. This study aimed to provide a comprehensive understanding of global diabetes prevention and control, reduce the risk of other diseases caused by diabetes, and provide reference data for relevant departments to formulate policies and measures related to the prevention and control of diabetes.

## Methods

### Study Data

This study was based on the GBD Study 2019 reported in 2020. Data arising from the GBD study was derived from the death registration system, vital registration, verbal autopsy, and mortality monitoring [27]. The reliability and stability of these data sources were demonstrated previously [11–13, 18]. Detailed data relating to the diabetes estimates used in this study can be found at https://vizhub.healthdata.org/gbd-results [11]. The data used for the APC model was acquired from two public websites (https://population.un.org/wpp/Download/Standard/CSV/ and https://seer.cancer.gov/stdpopulations/world.who.html/).

### Statistical Analysis

In this study, we first assessed the disease burden attributable to diabetes globally and by different subgroups in 2019. Then, we investigated the changing trend of disease burden of diabetes globally over the 25 years from 1990 to 2019. A linear regression model was used to calculate the estimated annual percentage change (EAPC) from 1990 to 2019. Furthermore, we predicted the number and age-standardized rates of diabetes-related incidence, prevalence, deaths, and DALYs from 2020 to 2044 using the APC model and the Bayesian APC model. Furthermore, we used the ARIMA model and ES model in time series models for sensitivity analysis.

Microsoft Office Excel 2019 (Los Angeles, CA, USA) and IBM SPSS 20.0 (Armonk, NY, USA) were used for database construction and data collation. R 4.0.2 was used for analysis. The “tidyverse” package was used to construct linear regression models to calculate EAPC values, the "nordpred" package was used to construct the APC model, the "BAPC" and “INLA” packages were used for BAPC models, and the "forecast" package was used to construct ARIMA and ES models for sensitivity analysis.

## Results

### The Disease Burden Attributable to Diabetes in 2019

The number of incidence cases related to diabetes in 2019 was 22239396 (95% uncertainty intervals (UI) 20599519–24058945) globally, the number of prevalence cases was 459875371 (95% UI 423474244–497980624), the number of deaths cases was 1551170 (95% UI 1445555–1650675), and the number of DALYs cases was 70880155 (95% UI 59707574–84174005). The corresponding age-standardized incidence rate (ASIR) was 267.54/100000 (95% UI 248.03/100000–289.18/100000), the age-standardized prevalence rate was 5555.39/100000 (95% UI:5118.84/100000–6013.77/100000), the age-standardized mortality rate (ASMR) was 19.47/100000 (95% UI 18.08/100000–20.71/100000), and the age-standardized DALYs rate was 858.96/100000 (95% UI 723.54/100000 -1019.86/100000) (Tables 1, 2, 3 and 4).

**Table 1:** The number of incidence cases and the ASIR of diabetes in 1990 and 2019, and its trends from 1990 to 2019 globally

Characteristics	1990		2019		1990–2019
	Number of incidence cases (95% UI)	ASIR/100000 (95% UI)	Number of incidence cases (95% UI)	ASIR/100000 (95% UI)	EAPC (95% CI)
Global	8728076 (8104287–9428627)	190.13 (176.63–205.25)	22239396 (20599519–24058945)	267.54 (248.03–289.18)	1.25 (1.19–1.31)
Gender					
Female	4260588 (3962871–4598733)	184.63 (171.16–199.49)	10658913 (9858272–11535020)	253.37 (234.55–273.62)	1.17 (1.10–1.24)
Male	4467488 (4135643–4831293)	195.77 (181.60–211.36)	11580483 (10723635–12524716)	281.68 (261.66–304.05)	1.32 (1.26–1.37)
Age					
Young groups (< 20 years)	466855 (357773–596862)	20.53 (15.74–26.25)	784051 (594679–1008637)	30.40 (23.06–39.11)	1.82 (1.68–1.96)
Middle-aged groups (20–59 years)	6117612 (5320441–6998782)	735.35 (604.22–888.99)	15880652 (13806332–18109567)	1104.10 (911.76–1326.68)	1.54 (1.44–1.63)
Elderly groups (≧60 years)	2143609 (1687535–2626532)	1357.21 (1058.93–1668.71)	5574693 (4406668–6845092)	1726.34 (1361.10–2124.30)	0.98 (0.84–1.12)
Type					
Type 1 diabetes	316765 (258425–387437)	5.57 (4.59–6.77)	569452 (463259–697407)	7.60 (6.18–9.32)	1.15 (1.10–1.20)
Type 2 diabetes	8411311 (7787867–9113457)	184.55 (170.91–199.70)	21669944 (20020895–23513486)	259.94 (240.35–281.44)	1.25 (1.19–1.31)
SDI regions					
High SDI	1909523 (1776355–2051810)	204.63 (189.92–219.60)	4089940 (3775798–4413582)	303.78 (281.09–326.28)	1.72 (1.56–1.87)
High-middle SDI	2078953 (1926951–2244451)	180.78 (167.89–194.57)	4417917 (4080270–4787797)	239.95 (222.48–259.21)	1.17 (1.03–1.32)
Middle SDI	2655736 (2452643–2890126)	196.74 (181.71–213.85)	7231808 (6704678–7840908)	267.32 (249.11–289.00)	1.01 (0.96–1.06)
Low-middle SDI	1508267 (1385641–1645787)	187.85 (173.85–204.83)	4656844 (4288359–5077940)	284.64 (262.33–309.60)	1.33 (1.29–1.37)
Low SDI	569906 (522181–621061)	176.91 (162.68–192.52)	1827175 (1673654–1995421)	244.14 (224.64–267.27)	1.04 (1.01–1.07)

*ASIR* age-standardized incidence rate

**Table 2:** The number of prevalence cases and the age-standardized prevalence rate of diabetes in 1990 and 2019, and its trends from 1990 to 2019 globally

Characteristics	1990		2019		1990–2019
	Number of prevalence cases (95% UI)	The age-standardized prevalence rate/100000 (95% UI)	Number of prevalence cases (95% UI)	The age-standardized prevalence rate/100000(95% UI)	EAPC(95% CI)
Global	158800093 (145974031–172491012)	3758.35 (3457.45–4075.26)	459875371 (423474244–497980624)	5555.39 (5118.84–6013.77)	1.46 (1.40–1.52)
Gender					
Female	79333553 (72820779–85965219)	3597.64 (3308.42–3894.72)	222003551 (203645130–240400977)	5168.86 (4748.06–5600.73)	1.36 (1.30–1.42)
Male	79466540 (72903579–86683298)	3932.91 (3617.30–4268.15)	237871820 (219394426–258012572)	5970.35 (5514.62–6462.83)	1.56 (1.50–1.61)
Age					
Young groups (< 20 years)	2792110 (2258827–3433949)	122.81 (99.35–151.04)	4574287 (3666007–5674569)	177.35 (142.13–220.01)	1.40 (1.35–1.45)
Middle-aged groups (20–59 years)	86572914 (76788820–97266864)	12705.35 (11305.52–14201.40)	234133469 (209261736–261541807)	19,571.94 (17,568.34–21,701.76)	1.58 (1.53–1.63)
Elderly groups (≧60 years)	71776658 (65137501–78755153)	43706.17 (39614.95–48003.21)	221167614 (201909294–241349830)	62,901.76 (57,335.17–68,731.55)	1.40 (1.31–1.49)
Type					
Type 1 diabetes	10345599 (8325510–12653666)	211.81 (171.58–257.60)	21968799 (17480371–27070679)	272.54 (216.98–336.95)	0.96 (0.90–1.01)
Type 2 diabetes	148454494 (135461386–162602314)	3546.54 (3243.75–3862.74)	437906572 (402043333–477018182)	5282.85 (4853.59–5752.09)	1.49 (1.43–1.55)
SDI regions					
High SDI	39538495 (36599269–42549538)	3981.41 (3680.63–4278.81)	97392510 (90171263–104542516)	6108.60 (5666.00–6556.10)	1.84 (1.68–1.99)
High-middle SDI	39357560 (36148129–42586348)	3571.66 (3285.09–3858.59)	98570355 (90489715–107132818)	5026.67 (4621.17–5468.08)	1.42 (1.30–1.55)
Middle SDI	45077664 (41179426–49538972)	3868.66 (3545.08–4230.75)	143920620 (132663860–156434065)	5515.18 (5091.51–5976.31)	1.23 (1.19–1.28)
Low-middle SDI	25661494 (23276745–28287884)	3696.20 (3367.04–4046.75)	88949990 (81217671–97616489)	5970.30 (5462.00–6512.44)	1.51 (1.46–1.56)
Low SDI	9065615 (8227325–10048306)	3244.24 (2949.46–3568.48)	30737282 (27775244–33953676)	4918.83 (4460.34–5414.76)	1.31 (1.27–1.35)

**Table 3:** The number of deaths cases and the ASMR of diabetes in 1990 and 2019, and its trends from 1990 to 2019 globally

Characteristics	1990		2019		1990–2019
	Number of deaths cases (95% UI)	ASMR/100000 (95% UI)	Number of deaths cases (95% UI)	ASMR/100000 (95% UI)	EAPC (95% CI)
Global	661824 (628054–695170)	17.92 (16.89–18.82)	1551170 (1445555–1650675)	19.47 (18.08–20.71)	0.19 (0.09–0.28)
Gender					
Female	363328 (337608–387306)	17.68 (16.39–18.91)	796081 (720072–859795)	18.21 (16.48–19.66)	0.00 (-0.09–0.09)
Male	298497 (284137–313989)	18.25 (17.24–19.22)	755089 (704658–809312)	20.98 (19.53–22.53)	0.38 (0.27–0.50)
Age					
Young groups (< 20 years)	9729 (7766–11203)	0.43 (0.34–0.49)	8703 (7500–10137)	0.34 (0.29–0.39)	− 0.91 (− 0.99–0.84)
Middle-aged groups (20–59 years)	163010 (153700–173490)	35.16 (33.23–37.36)	326631 (303414–351137)	38.57 (35.93–41.30)	0.24 (0.14–0.34)
Elderly groups (≧60 years)	489086 (458475–514430)	281.95 (264.83–296.41)	1215836 (1105108–1300295)	314.47 (287.41–336.18)	0.30 (0.22–0.39)
Type					
Type 1 diabetes	55417 (44066–63920)	1.23 (0.97–1.43)	78236 (68335–93806)	0.98 (0.85–1.17)	-1.01 (-1.10–0.92)
Type 2 diabetes	606407 (573069–637508)	16.69 (15.70–17.55)	1472934 (1371940–1565860)	18.49 (17.18–19.66)	0.26 (0.17–0.36)
SDI regions					
High SDI	132807 (125238–136742)	12.66 (11.92–13.05)	195298 (177169–206373)	9.68 (8.91–10.17)	− 1.41 (-1.69–1.14)
High-middle SDI	140296 (134045–146144)	14.18 (13.40–14.80)	263468 (241996–281548)	13.22 (12.11–14.14)	-0.33 (-0.44–0.21)
Middle SDI	194422 (184256–204792)	21.33 (20.04–22.61)	566686 (527582–609750)	24.77 (22.94–26.67)	0.55 (0.50–0.60)
Low-middle SDI	125632 (113686–138823)	24.31 (21.87–26.91)	371802 (339554–402061)	30.47 (27.73–32.96)	0.77 (0.65–0.89)
Low SDI	67925 (60637–75330)	32.41 (28.83–35.75)	152341 (138578–168455)	33.50 (30.53–36.78)	0.10 (0.01–0.18)

*ASMR* age-standardized mortality rate

**Table 4:** The number of DALYs cases and the age-standardized DALYs rate of diabetes in 1990 and 2019, and its trends from 1990 to 2019 globally

Characteristics	1990		2019		1990–2019
	Number of DALYs cases (95% UI)	The age-standardized DALYs rate/100000 (95% UI)	Number of DALYs cases (95% UI)	The age-standardized DALYs rate/100000 (95% UI)	EAPC (95% CI)
Global	28586671 (24620250–33182011)	690.63 (594.99–800.28)	70880155 (59707574–84174005)	858.96 (723.54–1019.86)	0.71 (0.67–0.75)
Gender					
Female	14687600 (12569740–16999211)	671.86 (575.47–778.12)	34431769 (28926842–41081703)	797.07 (669.52–951.31)	0.55 (0.52–0.59)
Male	13899072 (11898419–16174620)	712.33 (611.76–826.31)	36448385 (30380549–43342627)	926.63 (775.70–1099.14)	0.86 (0.81–0.91)
Age					
Young groups (< 20 years)	918884 (744010–1056530)	40.42 (32.72–46.47)	915719 (779173–1072188)	35.50 (30.21–41.57)	-0.50 (-0.55–0.45)
Middle-aged groups (20–59 years)	12589079 (10548627–15015914)	2191.39 (1856.14–2585.38)	29863523 (24364292–36537696)	2867.38 (2369.31–3454.35)	0.89 (0.85–0.94)
Elderly groups (≧60 years)	15078708 (13059419–17404096)	9141.37 (7912.24–10561.00)	40100913 (33992040–47325382)	11,283.17 (9541.93–13,355.6)	0.70 (0.63–0.78)
Type					
Type 1 diabetes	3108573 (2558605–3571553)	62.30 (51.35–71.67)	4580404 (3905618–5382419)	57.41 (49.11–67.23)	-0.45 (-0.51–0.39)
Type 2 diabetes	25478098 (21701411–29776367)	628.33 (537.22–730.86)	66299751 (55477042–79005166)	801.55 (670.58–954.43)	0.81 (0.77–0.85)
SDI regions					
High SDI	5502915 (4609809–6508797)	544.35 (455.46–644.28)	10766841 (8499481–13431403)	644.23 (504.92–804.42)	0.53 (0.48–0.58)
High-middle SDI	6315935 (5337884–7454391)	582.59 (493.55–686.24)	12962949 (10519486–15769328)	650.08 (526.37–790.03)	0.39 (0.30–0.49)
Middle SDI	8671977 (7507099–9963570)	778.85 (677.06–895.96)	24669009 (21127433–28951517)	964.90 (829.09–1132.95)	0.72 (0.68–0.75)
Low-middle SDI	5398312 (4662543–6210923)	827.39 (718.19–946.21)	15851124 (13460490–18563465)	1117.75 (953.32–1304.21)	0.95 (0.91–1.00)
Low SDI	2670056 (2357153–3063747)	1008.51 (888.09–1150.52)	6565511 (5615632–7609802)	1143.21 (983.72–1322.19)	0.37 (0.32–0.41)

*DALY* disability-adjusted life years

**Fig. 1:**
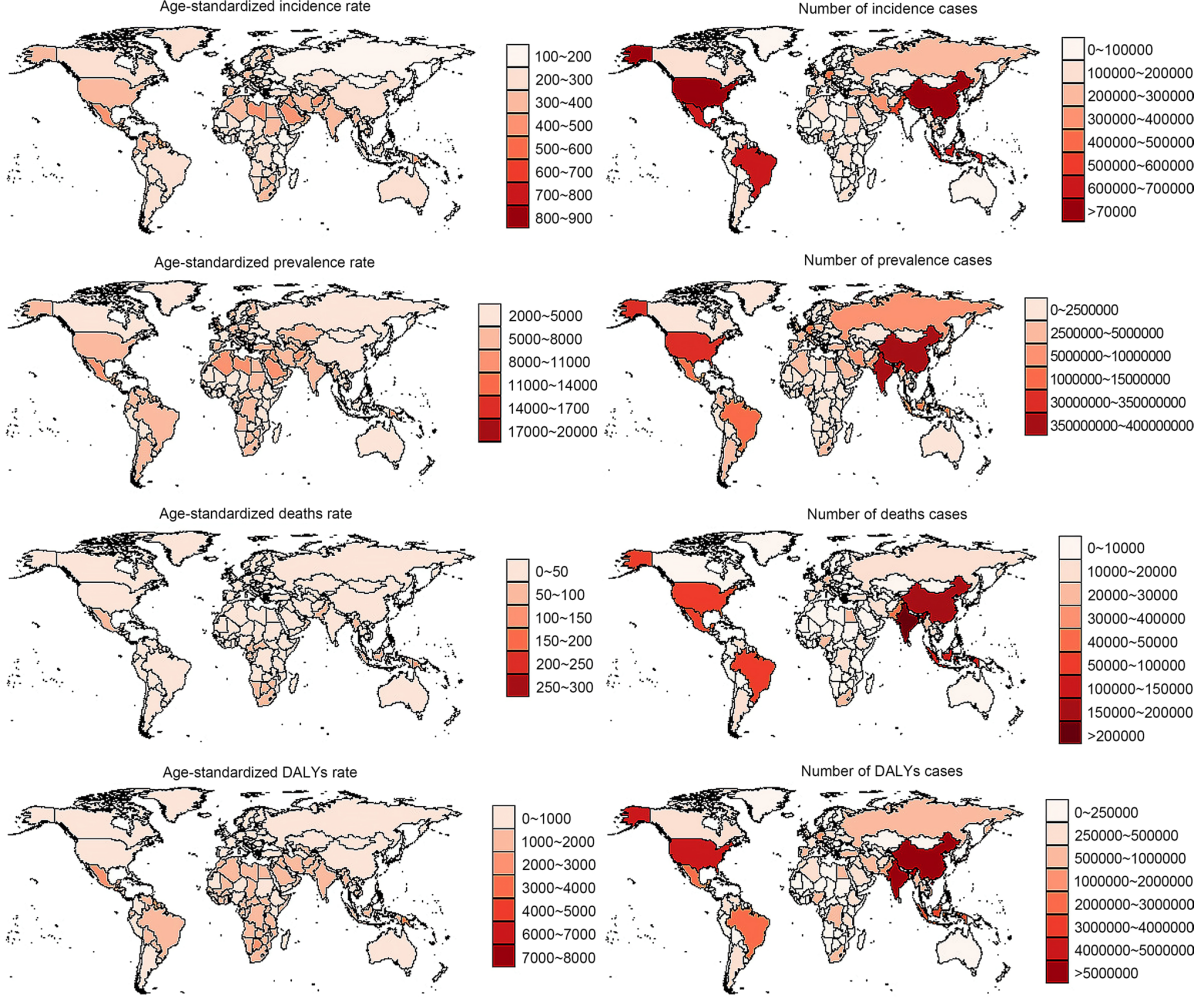
The global disease burden of diabetes across 195 countries and territories in 2019

For males, the number of incidence cases was 11580483 (95% UI 10723635–12524716), the number of prevalence cases was 237871820 (95% UI 95% UI 219394426–258012572) the number of deaths cases was 755089 (95% UI 704658–809312) and the number of DALYs cases was 36448385 (95% UI 30380549–43342627). The number of incidence cases for females was 10658913 (95% UI 9858272–11535020) the number of prevalence cases was 222003551 (95% UI 203645130–240400977) the number of deaths cases was 796081 (95% UI: 720072–859795) and the number of DALYs cases was 34431769 (95% UI 28926842–41081703). The number of incidence cases, the number of prevalence cases, and the number of DALYs cases for males were 1.09-fold, 1.07-fold, and 1.06-fold higher than that of females, respectively. The number of deaths cases was 1.05-fold higher in females than in males. The ASIR for males was 281.68/100000 (95% UI: 261.66/100000–304.05/100000), the age-standardized prevalence rate was 5970.35/100000 (95% UI 5514.62/100000–6462.83/100000), the ASMR was 20.98/100000 (95% UI 19.53/100000–22.53/100000), and the age-standardized DALYs rate was 926.63/100000 (95% UI: 775.70/100000–1099.14/100000). For females, the ASIR was 253.37/100000 (95% UI: 234.55/100000–273.62/100000), the age-standardized prevalence rate was 5168.86/100000 (95% UI 4748.06/100000–5600.73/100000), the ASMR was 18.21/100000 (95% UI 16.48/100000–19.66/100000), and the age-standardized DALYs rate was 797.07/100000 (95% UI 669.52/100000–951.31/100000). The ASIR, the age-standardized prevalence rate, the ASMR, and the age-standardized DALYs rates in males were 1.11-fold, 1.16-fold, 1.15-fold, and 1.16-fold higher than in females, respectively (Tables 1, 2, 3 and 4, Fig. S1). Therefore, males should be treated as the key population for early screening and the preventive control of diabetes. In addition to the number of incidence cases and the number of prevalence cases, the other indicators increased with increasing age, as observed in young groups (< 20 years old), middle-aged groups (20–59 years old), elderly groups (≧60 years-of-age). The number of incidence cases and the number of prevalence cases was highest in the middle-aged groups, followed by the elderly groups; the lowest values were in the young groups (Tables 1, 2, 3 and 4, Fig. S2). The disease burden associated with different types of diabetes varied significantly, with the disease burden attributable to type 2 diabetes being greater than type 1 diabetes. The number of incidence cases of type 1 diabetes was 569452 (95% UI 463259–697407) in 2019, the number of prevalence cases was 21968799 (95% UI 17480371–27070679), the number of deaths cases was 78236 (95% UI 68335–93806), and the number of DALYs cases was 4580404 (95% UI 3905618–5382419). The ASIR was 7.60/100000 (95% UI 6.18/100000–9.32/100000), the age-standardized prevalence rate was 272.54/100000 (95% UI 216.98/100000–336.95/100000), the ASMR was 0.98/100000 (95% UI 0.85/100000–1.17/100000), and the age-standardized DALYs rate was 57.41/100000 (95% UI 49.11/100000–67.23/100000). For type 2 diabetes, the number of incidence cases was 21669944 (95% UI 20020895–23513486) the number of prevalence cases was 437906572 (95% UI 402043333–477018182) the number of deaths cases was 1472934 (95% UI 1371940–1565860) and the number of DALYs cases was 66299751 (95% UI 55477042–79005166). The ASIR was 259.94/100000 (95% UI 240.35/100000–281.44/100000), the age-standardized prevalence rate was 5282.85/100000 (95% UI 4853.59/100000–5752.09/100000), the ASMR was 18.49/100000 (95% UI 17.18/100000–19.66/100000), and the age-standardized DALYs rate was 801.55/100000 (95% UI 670.58/100000–954.43/100000). The number of incidence cases, the number of prevalence cases, the number of deaths cases and the number of DALYs cases for type 2 diabetes was 38.05-fold, 19.93-fold, 18.83-fold, and 14.47-fold higher than that of type 1 diabetes, respectively. The ASIR, the age-standardized prevalence rate, the ASMR, and the age-standardized DALYs rates for type 2 diabetes were 34.20-fold, 19.38-fold, 18.87-fold, and 13.96-fold higher than that of type 1 diabetes, respectively (Tables 1, 2, 3 and 4, Fig. S3). The disease burden attributable to diabetes also differed across SDI regions. The diabetes-related ASIR and the age-standardized prevalence rate were highest in the high SDI regions, followed by the low-middle SDI regions and middle SDI regions. The diabetes-related ASIR and the age-standardized prevalence rate for high-middle SDI regions and low SDI regions were lower. The ASMR and the age-standardized DALYs rate attributable to diabetes decreased with as the SDI increased. The number of incidence cases, the number of prevalence cases, the number of deaths cases, and the number of DALYs cases first decreased with a decreased in SDI, reaching the highest in middle SDI regions, and then showed a downwards trend (Tables 1, 2, 3 and 4, Fig. S4). The disease burden attributable to diabetes differed across countries, as shown in Fig. 1.

### The Trend of Disease Burden of Diabetes from 1990 to 2019

The disease burden attributable to diabetes showed significant increases from 1990 to 2019 globally. The number of incidence cases of diabetes-related cases increased from 8728076 (95% UI: 8104287–9428627) in 1990 to 22239396 (95% UI: 20599519–24058945) in 2019; the corresponding ASIR increased from 190.13 (95% UI 176.63–205.25) in 1990 to 267.54 (95% UI 248.03–289.18) in 2019, with an EAPC of 1.25 (95% confidence interval (CI) 1.19–1.31). The number of prevalence cases of diabetes-related cases increased by 189.59%, increasing from 158,800,093 (95% UI 145974031–172,491,012) in 1990 to 459875371 (95% UI 423474244–497980624) in 2019. The age-standardized rate (per 100,000 population) of prevalence increased from 3758.35 (95% UI 3457.45–4075.26) in 1990 to 5555.39 (95% UI 5118.84–6013.77) in 2019, with an EAPC of 1.46 (95% CI 1.40–1.52). The diabetes-related number of deaths cases increased from 661824 (95% UI: 628054–695170) in 1990 to 1551170 (95% UI: 144555–1650675) in 2019, the ASMR increased from 17.92 (95% UI 16.89–18.82) in 1990 to 19.47 (95% UI 18.08–20.71) in 2019, with an EAPC of 0.19 (95% CI 0.09–0.28). Globally, the number of DALYs cases of diabetes increased from 28586671 (95% UI 24620250–33182011) in 1990 to 70880155 (95% UI 59707574–84174005) in 2019. The corresponding age-standardized DALYs rate increased from 690.63 (95% UI 594.99–800.28) to 858.96 (95% UI 723.54–1019.86) in 2019 during this period, with an EAPC of 0.71 (95% CI 0.67–0.75) (Tables 1, 2, 3 and 4).

In subgroup analysis, the disease burden attributable to diabetes increased from 1990 to 2019 in both genders. The increase in disease burden of diabetes for males was more significant than that of females (Tables 1, 2, 3 and 4, Fig. S5). From 1990 to 2019, the disease burden attributable to diabetes increased in middle-aged groups and elderly groups. The disease burden remained relatively stable and was consistently lower in the young groups over the last 30 years (Tables 1, 2, 3 and 4, Fig. S6). There were differences in disease burden between the different types of diabetes. Except for the ASMR and the age-standardized DALYs rate attributable to type 1 diabetes, other indicators of type 1 diabetes, and all indicators attributable to type 2 diabetes, increased. Moreover, the rising trend of type 2 diabetes was more significant than for type 1 diabetes (Tables 1, 2, 3 and 4, Fig. S7). From 1990 to 2019, the diabetes-related number of incidence cases, deaths cases, and DALYs cases increased in all regions, regardless of the level of SDI. The ASIR, the age-standardized prevalence rate, and the age-standardized DALYs rate increased in different SDI regions. From 1990 to 2019, the ASMR increased in all SDI regions except for high-SDI regions and high-middle SDI regions (Tables 1, 2, 3 and 4, Fig. S8). Furthermore, we analyzed the relevant influencing factors associated with diabetes in all SDI regions. Analysis showed that some factors were associated with diabetes, such as alcohol use, ambient particulate matter pollution, high body-mass index, high fasting plasma glucose, high intake of processed meat, high intake of red meat, high intake of sugar-sweetened beverages, high temperature, household solid fuels/air pollution, low levels of fiber, low intake of fruits, low intake of nuts and seeds, low intake of whole grains, low levels of physical activity, low temperature, secondhand smoke. Of these contributing factors, high fasting plasma glucose had the largest effect, followed by high body-mass index (Fig. S9). By geographic regions, the trends in disease burden attributable to diabetes differed across countries (Tables 1, 2, 3 and 4, Fig. S10).

### Predictions of the Disease Burden of Diabetes

Our predicted results showed that all disease burden indicators except for the ASMR for both genders would increase in the next 25 years. The ASMR for males is expected to increase in the same period while the ASMR for females is expected to decrease **(**Fig. S11 and Fig. S12**)**. The same results were obtained from the BAPC model. All disease burden of diabetes indicators except for the age-standardized DALYs rate for both genders is expected to increase. However, the age-standardized DALYs rate for females is expected to decrease while the age-standardized DALYs rate for males is expected to increase **(**Fig. S13 and Fig. S14**)**. Sensitivity analysis also verified the stability of these results (Fig. S15 to Fig. S18).

## Discussion

This study collected global disease burden of diabetes data from the GBD 2019 database from 1990 to 2019. Then, we comprehensively assessed and predicted the situation and changes in the disease burden attributable to diabetes globally. Our results showed that diabetes caused a serious burden globally in 2019, the disease burden attributable to diabetes worldwide has increased over the past 30 years, and the disease burden is expected to increase in the future. This study found that the disease burden attributable to diabetes in men was more serious than in women. The disease burden attributable to diabetes was high in the middle-aged and elderly populations. The disease burden associated with type 2 diabetes was relatively higher than that for type 1 diabetes. Data suggested that the health management of type 2 diabetes patients and high-risk groups should be strengthened, and the awareness of prevention should be improved to reduce the disease burden of diabetes. Furthermore, males, middle-aged, and elderly subjects should allocate appropriate health resources as the focus of diabetes. Finally, this study also found that the disease burden attributable to diabetes varied in different SDI regions and different countries, thus facilitating detection in high-risk areas.

The overall disease burden of diabetes was higher in men than in women. The diabetes-related number of deaths cases in females was higher than that in males, but all other indicators were lower in women than in men. Furthermore, the increase in disease burden of diabetes for men was more significant than that of women. Therefore, males should be treated as a high-risk group for diabetes, and we should pay attention to males with this respect. Differences in the gender distribution of disease burden of diabetes have been related to physiological and metabolic differences between males and females [28]. These differences might also be related to the higher level of exposure to risk factors such as excessive alcohol consumption, obesity, sedentary sitting, smoking, and excessive weight gain in the male population [29–31]. In addition, these differences were also closely related to educational level, socioeconomic, and cultural factors [32–34]. For example, females have been shown to be concerned about disease including diabetes, had higher compliance with treatment, and had a stronger awareness of diabetes-related self-management [35].

Our analysis showed that disease burden increased with age. The disease burden attributable to diabetes increased in middle-aged groups and elderly groups from 1990 to 2019. Therefore, age represents a risk factor for diabetes; this finding was consistent with the findings of most previous studies [36–38]. These findings indicated that the disease burden gradually increased with age growth. This suggests that the elderly should be a significant focus of diabetes. We should strengthen health monitoring and health publicity for the elderly, improve health awareness of regular blood glucose tests, achieve early detection, early management, and early treatment for high-risk groups such as those with high blood sugar, excessive weight and obesity, and reduce the disease burden of diabetes.

With regards to the subtypes of diabetes, the disease burden attributable to type 2 diabetes was greater than that for type 1 diabetes in 2019. Except for the ASMR and the age-standardized DALYs rate attributable to type 1 diabetes, other indicators of type 1 diabetes and all indicators attributable to type 2 diabetes are all increasing. Moreover, the rising trend of type 2 diabetes was more significant than that for type 1 diabetes. This might be due to the different pathophysiology and the different treatment methods of the two subtypes of diabetes. Type 1 diabetes is mostly dependent on insulin therapy. The improvement of insulin pump application and the strengthening of diabetes management education all contributed to improving the disease burden attributable to type 1 diabetes [39, 40]. However, type 2 diabetes was closely related to metabolic factors and behavioral habits and was affected by the level of social and economic development [41, 42]. Over recent years, people’s habits of living and eating have changed. Changes in these risk factors contributed to an increased disease burden of type 2 diabetes.

The disease burden attributable to diabetes varied across different SDI regions. SDI is a comprehensive index to measure a country’s socio-economic development level, and is composed of fertility rate, resident income, and educational level. SDI levels are known to be strongly associated with health outcomes [43]. We found that the diabetes-related ASIR and the age-standardized prevalence rate were highest in high SDI regions. The ASMR and the age-standardized DALYs rate attributable to diabetes decreased as SDI increased and were highest in low SDI regions. In economically developed countries, there were sufficient medical resources. The screening system for diabetes is more universal; thus, the early diagnostic rate of diabetes was higher. Furthermore, the diagnostic techniques for type 1 diabetes and type 2 diabetes are better. These factors are why the incidence and prevalence rate of diabetes were high in developed regions. In the United States, a 2012 report showed that the proportion of Americans with undiagnosed diabetes was approximately 28%, and the proportion of patients with no awareness of diabetes was approximately 28% [44]. In India, a study from 2015 to 2016 showed that more than 50% of diabetic patients aged 15–50 had not been diagnosed, and approximately 42% had no awareness of diabetes [45]. Timely and standardized intervention treatment provided diabetic patients with a good prognosis, low mortality, and overall low DALYs. Therefore, the diabetes-related mortality and DALYs rate was lower in developed countries. Although the incidence and prevalence of diabetes were low in economically less developed countries, the lack of access to drugs (especially insulin, which is essential for type 1 diabetes) and surveillance technologies in these countries led to high rates of mortality and disability [46]. The IDF report noted that basal insulin was provided to children by governments in 75% of high-income countries and 50% of middle-income countries, but not in low-income countries. In high-income countries, 81% and 84% of subjects could receive short-acting and intermediate-acting insulin, 46% and 44% of subjects in middle-income countries could receive short-acting and intermediate-acting insulin, while only one low-income country provides insulin. The basic diabetes medication metformin was available in 20% of low-income countries, 64% of middle-income countries, and 88% of high-income countries [47]. Therefore, it is very important to strengthen the supply of basic insulin and drugs for diabetes in economically underdeveloped areas.

The distribution of disease burden of diabetes varied across countries. In general, the overall disease burden was highest in large countries with large populations. The number of cases was relatively high in Asia. After adjusting for confounding variables, the disease burden was heaviest in Africa and South Asia. In most developing countries, the disease burden attributable to diabetes was increasing. In India, the DALYs rate of diabetes increased by about 39.6% from 1990 to 2016, and showed the largest increased of all non-communicable diseases [48]. In the Middle East, the diabetes-related DALYs cases increased from 2.285 million to 6.795 million between 1990 and 2015, with a growth rate of 197.4% [49]. In Brazil, the DALYs increased by 58.3% from 1990 to 2015 [50].

This study had some limitations that need to be considered. First, the risk factors such as population, environment, health, and economy associated with diabetes were not included in this study due to the absence of such data in the database. Therefore, we should address this shortfall in the updated database. Second, the database only classified diabetes into type 1 and type 2; therefore, we were unable to consider other types of diabetes, such as gestational diabetes.

## Conclusion

Diabetes has caused a significant disease burden globally, and the disease burden of diabetes increased significantly globally from 1990 to 2019. The results of our predictive models showed that the disease burden will continue to grow in the future if there is no effective intervention. This study analyzed the global distribution of diabetes disease burden to provide a reference for making prevention and control decisions and resource allocation.

## Supplementary Information

Below is the link to the electronic supplementary material.

Supplementary file1 (DOCX 11643 KB)

## Data Availability

The datasets analyzed during the current study are publicly available.

## Abbreviations

DALYs: Disability-adjusted life years
IDF: International diabetes federation
YLL: Years of life lost
YLD: Years lived with disability
GBD: Global burden of disease
APC: Age-period-cohort
ARIMA: Auto-regressive integrated moving average model
ES: Exponential smoothing model
EAPC: Estimated annual percentage change
UI: Uncertainty intervals
ASIR: Age-standardized incidence rate
ASMR: Age-standardized mortality rate
CI: Confidence interval
