# Supplementary material for: The Description and Prediction of Incidence, Prevalence, Mortality, Disability-Adjusted Life Years Cases, and Corresponding Age-Standardized Rates for Global Diabetes
Source: J Epidemiol Glob Health. 2023 Jul 3;13(3):566–76. doi: 10.1007/s44197-023-00138-9 (PMC10469163; doi:10.1007/s44197-023-00138-9)
Supplement: Supplementary file 1 — Supplementary file1 (DOCX 11643 KB) [file 44197_2023_138_MOESM1_ESM.docx]

**Figure legends**

**Figure S1** The global disease burden of diabetes by sex in 2019

**Figure S2** The global disease burden of diabetes across different age groups in 2019

**Figure S3** The global disease burden of diabetes for different subtypes in 2019

**Figure S4** The global disease burden of diabetes across different SDI regions in 2019. **Abbreviation:** SDI: socio-demographic index

**Figure S5** Trends in the disease burden of diabetes globally by sex from 1990 to 2019

**Figure S6** Trends in the disease burden of diabetes globally by age from 1990 to 2019

**Figure S7** Trends in the disease burden across different subtypes of diabetes globally from 1990 to 2019

**Figure S8** Trends in the disease burden of diabetes in different SDI regions globally from 1990 to 2019. **Abbreviation:** SDI: socio-demographic index

**Figure S9** The relevant influencing factors associated with diabetes in all SDI regions. **Abbreviation:** SDI: socio-demographic index

**Figure S10** The EAPC value of diabetes ASR from 1990 to 2019. **Abbreviation:** EAPC: estimated annual percentage change; ASR: age-standardized rate

**Figure S11** Trends in diabetes-related ASIR (a and b), the age-standardized prevalence rate (c and d), ASMR (e and f), and age-standardized DALYs rate (g and h) by sex globally from 1990 to 2044: observed (solid lines) and predicted rates of the APC model (dashed lines). **Abbreviations:** ASIR, age-standardized incidence rate; ASMR, age-standardized mortality rate; DALY: disability-adjusted life years; APC: age-period-cohort

**Figure S12** Trends in the number of incidence cases (a and b), the number of prevalence cases (c and d), number of deaths cases (e and f), and number of DALYs cases (g and h) attributable to diabetes by sex globally from 1990 to 2044: observed (before 2019) and predicted (after 2019) numbers of the APC model. Shading indicates if the rate remained stable (baseline reference), decreased by 1% per year (optimistic reference, lower limit), or increased by 1% per year (pessimistic reference, upper limit) based on the observed rate in 2019. Three methods were used in the prediction. The red line is calculated by the predicted rate of each 5-year group and average population size of the 5-year group. The blue line method was used to calculate the rate of each group in terms of the predicted rate for each 5-year group and average population situation of the 5-year groups. The yellow line is calculated by the predicted rate of each 5-year group and the annual population situation. **Abbreviations:** DALY: disability-adjusted life years; APC: age-period-cohort

**Figure S13** Trends in diabetes-related ASIR (a and b), the age-standardized prevalence rate (c and d), ASMR (e and f), and age-standardized DALYs rate (g and h) by sex globally from 1990 to 2044: observed (dashed lines) and predicted rates of the BAPC model (solid lines). The blue region shows the upper and lower limits of the 95% UIs. **Abbreviations:** ASIR, age-standardized incidence rate; ASMR, age-standardized mortality rate; DALY, disability-adjusted-life-year; BAPC: Bayesian age-period-cohort; UIs: uncertainty intervals

**Figure S14** Trends in the number of incidence cases (a and b), the number of prevalence cases (c and d), number of deaths cases (e and f), and number of DALYs cases (g and h) attributable to diabetes by sex globally from 1990 to 2044: observed (before 2019) and predicted (after 2019) numbers of the BAPC model. Shading indicates if the rate remained stable (baseline reference), decreased by 1% per year (optimistic reference, lower limit), or increased by 1% per year (pessimistic reference, upper limit) based on the observed rate in 2019. The curve formed by the triangle represents the prediction result of the BAPC model. **Abbreviations:** DALY, disability-adjusted-life-year; BAPC: Bayesian age-period-cohort

**Figure S15** Trends in diabetes-related ASIR (a and b), the age-standardized prevalence rate (c and d), ASMR (e and f), and age-standardized DALYs rate (g and h) by sex globally from 1990 to 2044: observed (before 2019) and predicted rates of the ARIMA model (after 2019). Shading indicates the upper and lower limits of the 95% CIs. **Abbreviations:** ASIR, age-standardized incidence rate; ASMR, age-standardized mortality rate; DALYs, disability-adjusted-life-years; ARIMA, autoregressive integrated moving average; CIs, confidence intervals

**Figure S16** Trends in the number of incidence cases (a and b), the number of prevalence cases (c and d), number of death cases (e and f), and number of DALYs cases (g and h) attributable to diabetes by sex globally from 1990 to 2044: observed (before 2019) and predicted numbers of the ARIMA model (after 2019). Shading indicates the upper and lower limits of the 95% CIs. **Abbreviations:** DALY, disability-adjusted-life-years; ARIMA, autoregressive integrated moving average; CIs, confidence intervals

**Figure S17** Trends in diabetes-related ASIR (a and b), the age-standardized prevalence rate (c and d), ASMR (e and f), and age-standardized DALYs rate (g and h) by sex globally from 1990 to 2044: observed (before 2019) and predicted rates of the ES model (after 2019). Shading indicates the upper and lower limits of the 95% CIs. **Abbreviations:** ASIR, age-standardized incidence rate; ASMR, age-standardized mortality rate; DALY, disability-adjusted-life-years; ES, exponential smoothing; CIs, confidence intervals

**Figure S18** Trends in the number of incidence cases (a and b), the number of prevalence cases (c and d), number of death cases (e and f), and number of DALYs cases (g and h) attributable to diabetes by sex globally from 1990 to 2044: observed (before 2019) and predicted numbers of the ES model (after 2019). Shading indicates the upper and lower limits of the 95% CIs. **Abbreviations:** DALY, disability-adjusted-life-years; ES, exponential smoothing; CIs, confidence interval

**
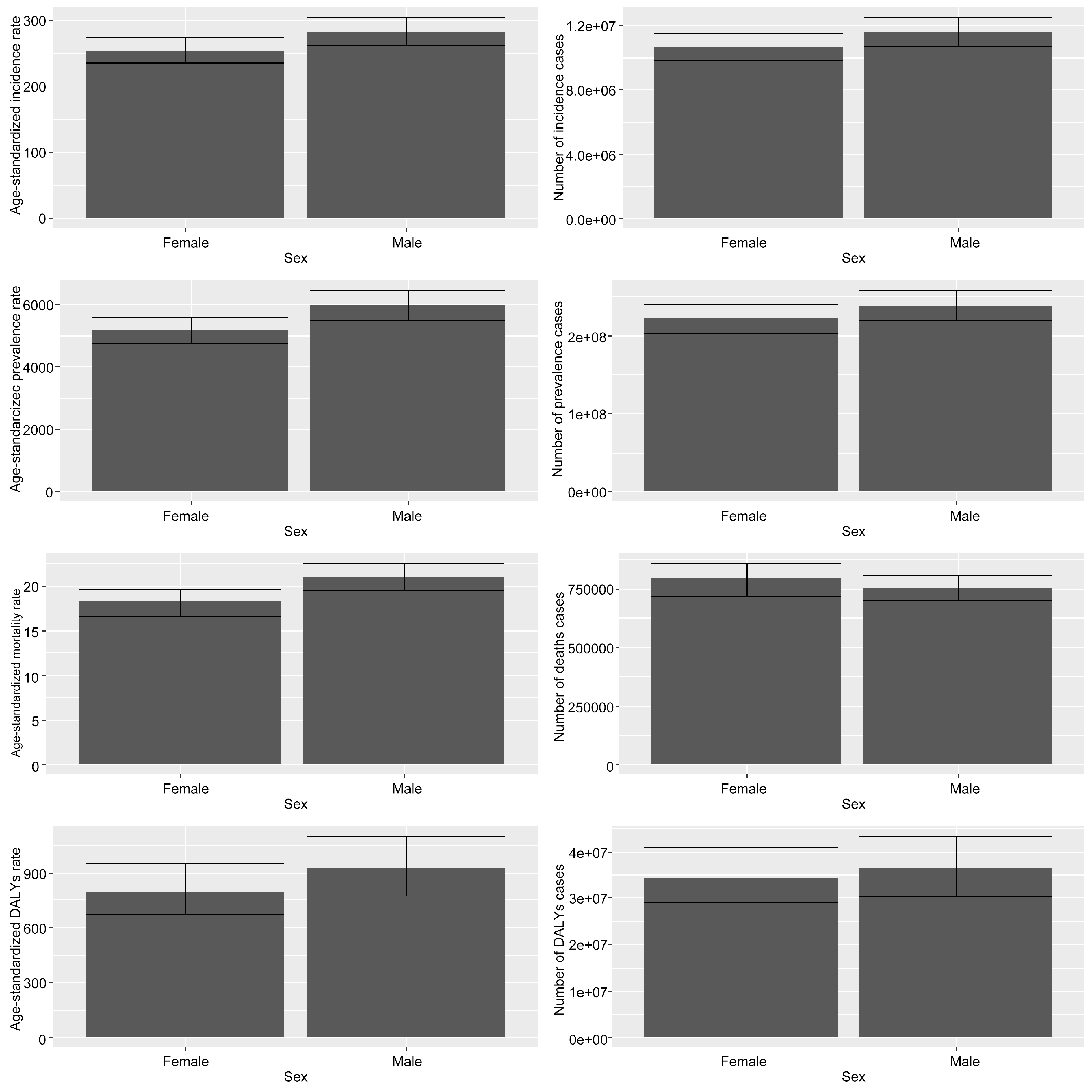
**

**Figure S1.** The global disease burden of diabetes by sex in 2019


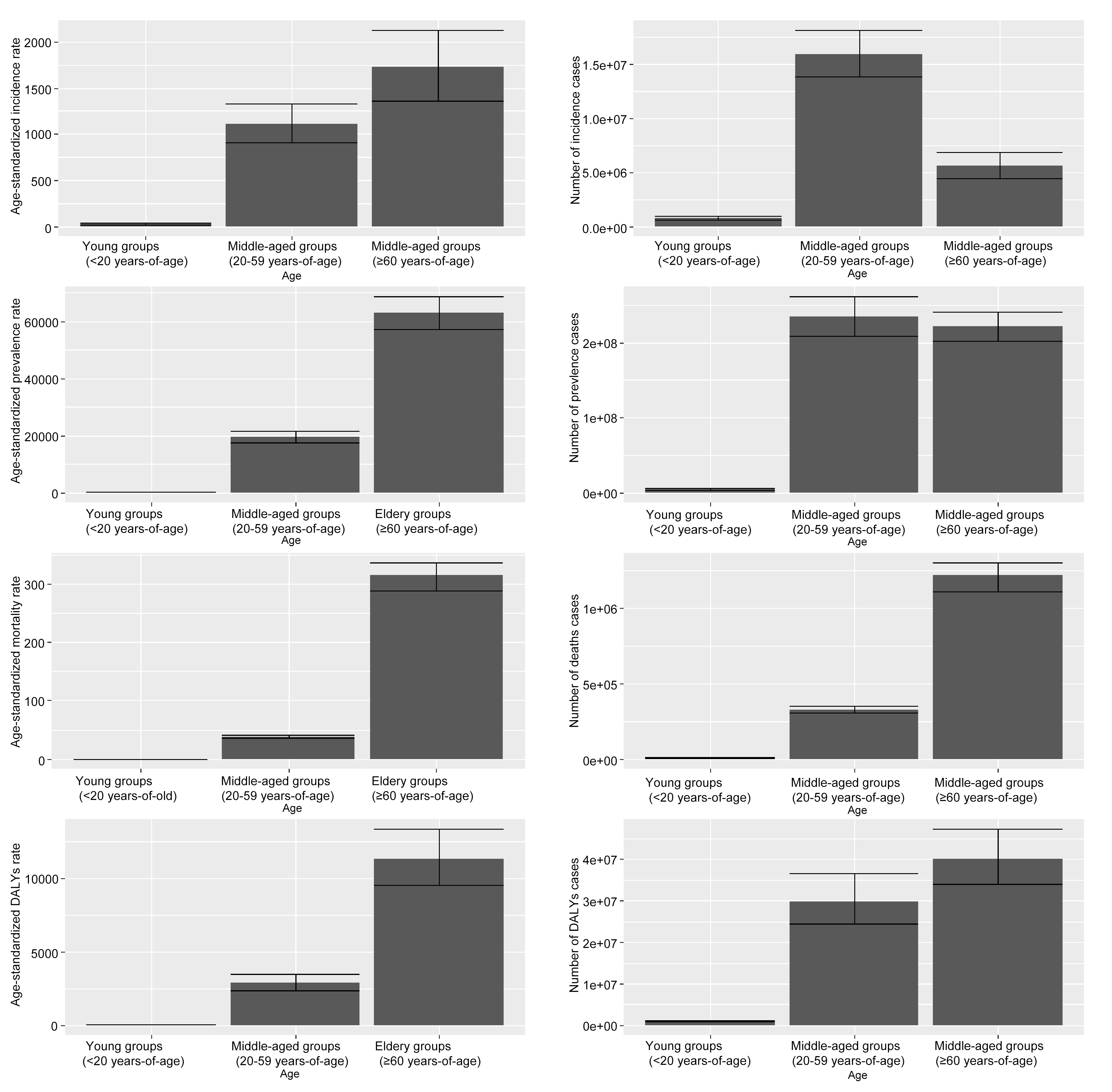


**Figure S2.** The global disease burden of diabetes across different age groups in 2019


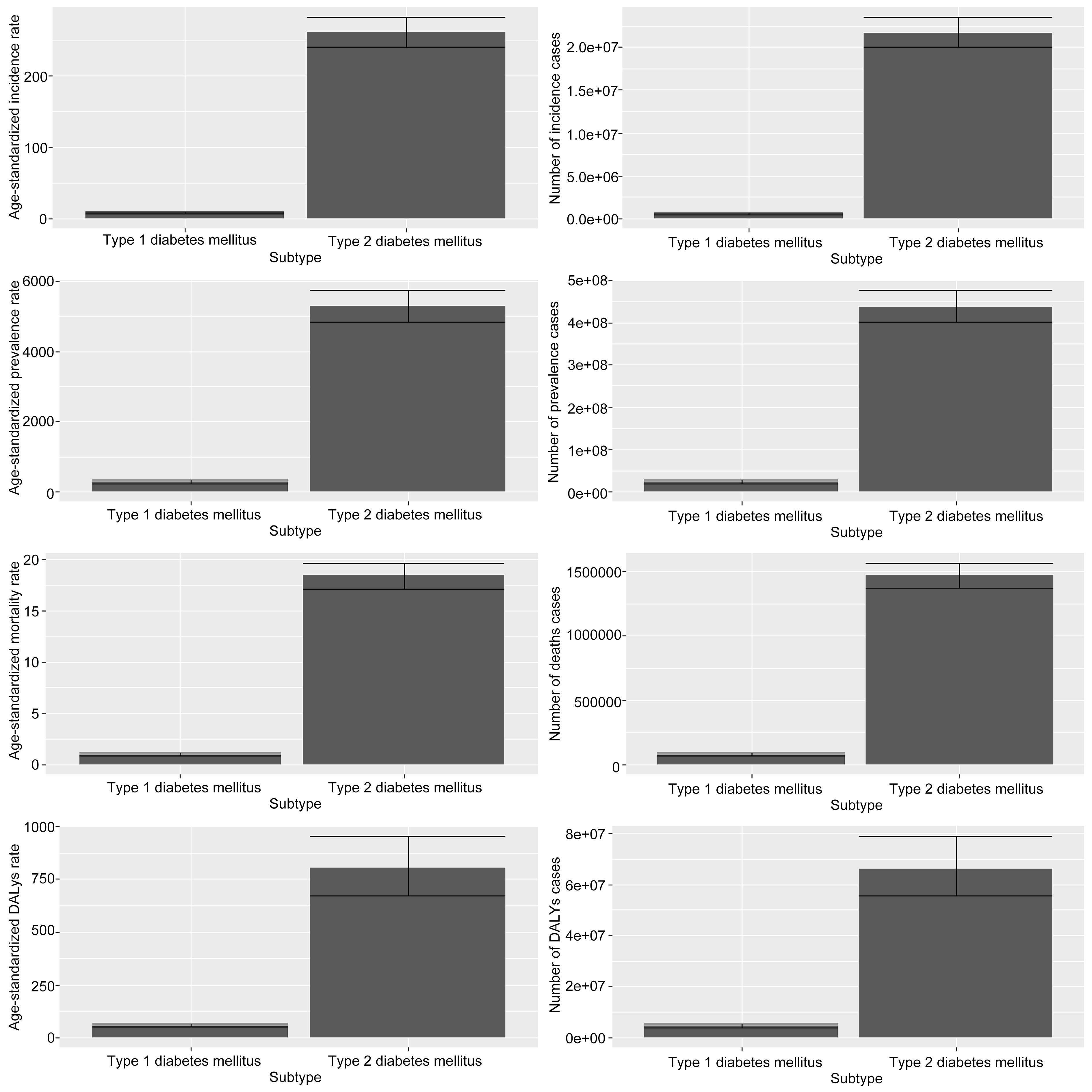


**Figure S3.** The global disease burden of diabetes for different subtypes in 2019


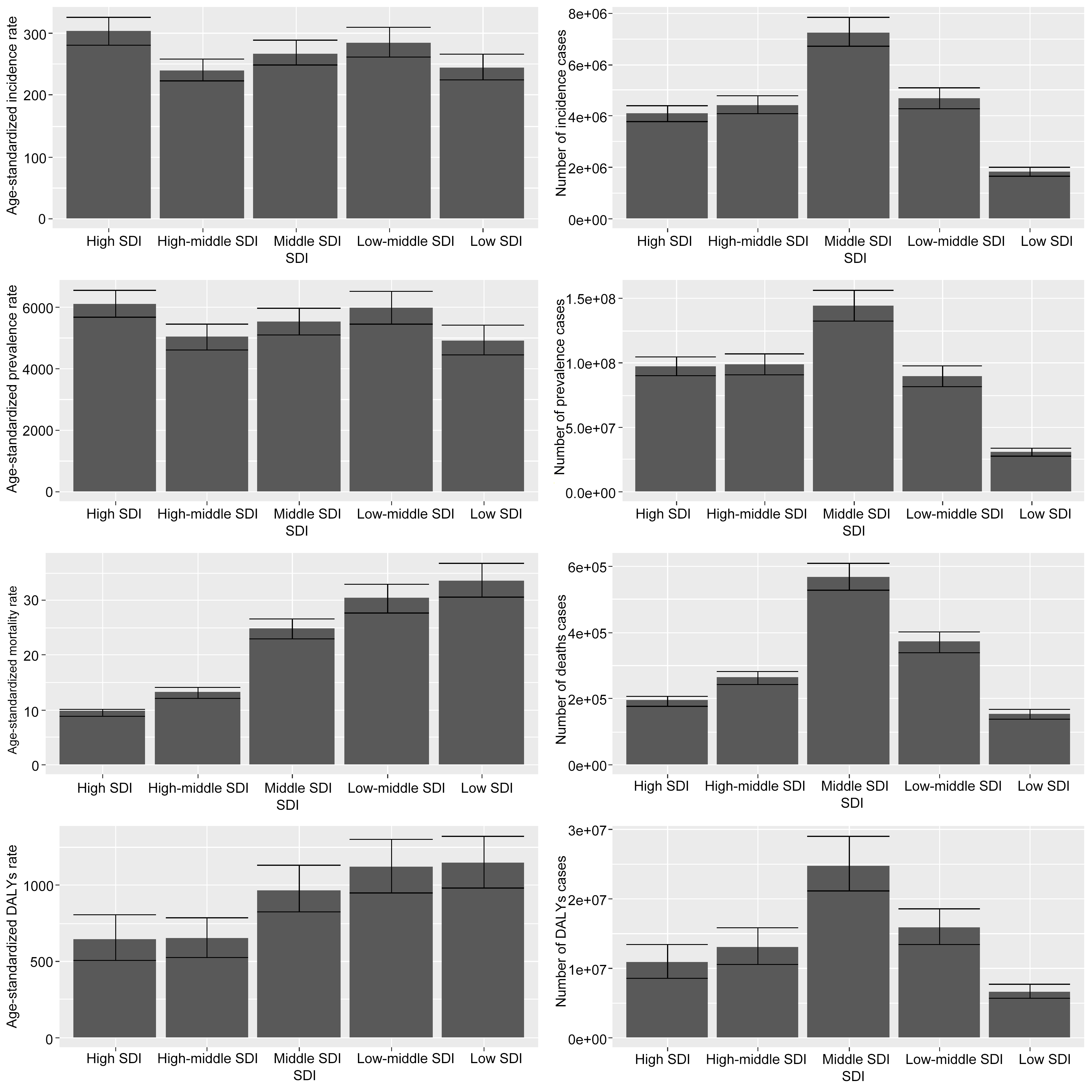


**Figure S4.** The global disease burden of diabetes across different SDI regions in 2019. **Abbreviation:** SDI: socio-demographic index

**
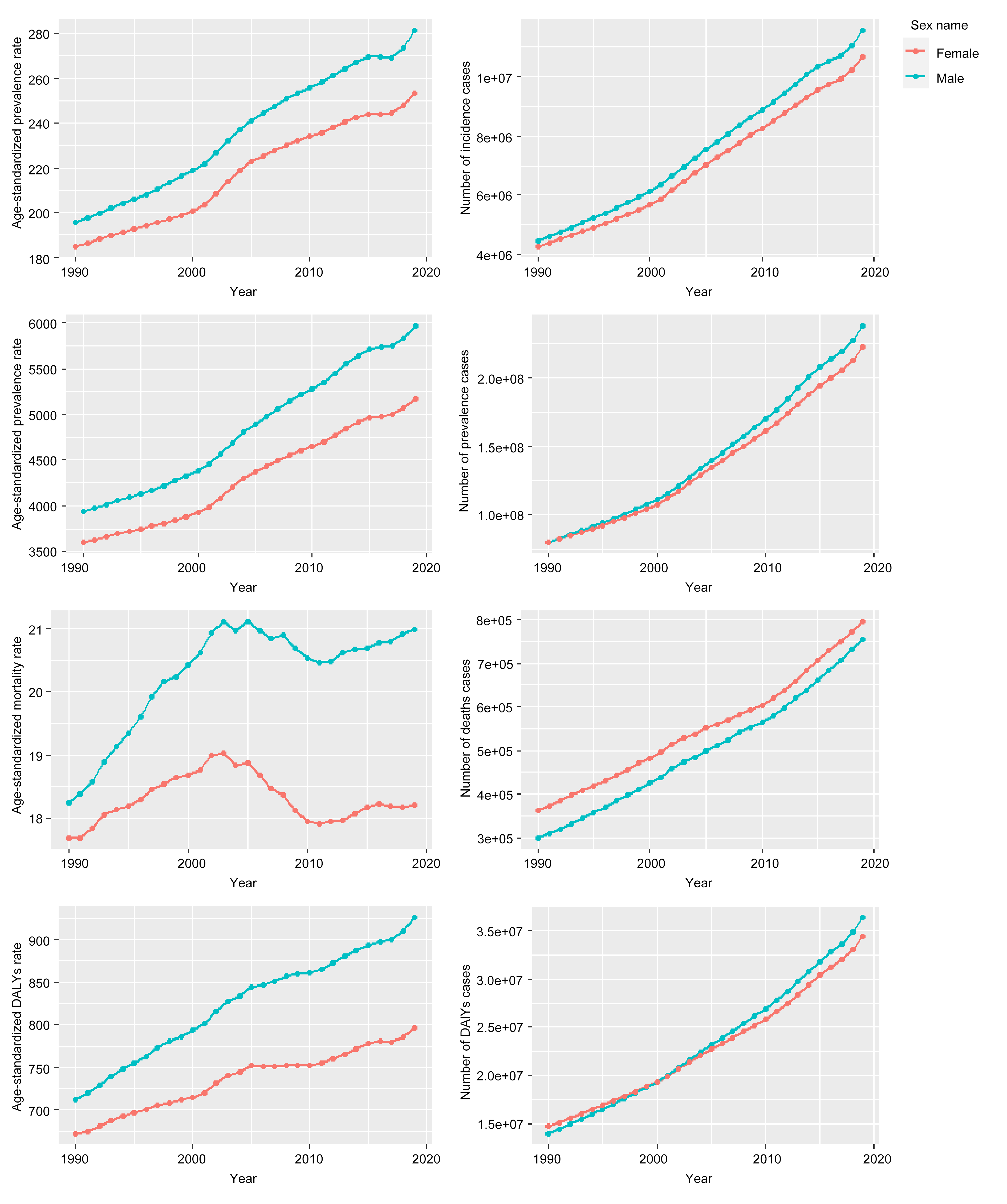
**

**Figure S5.** Trends in the disease burden of diabetes globally by sex from 1990 to 2019

**
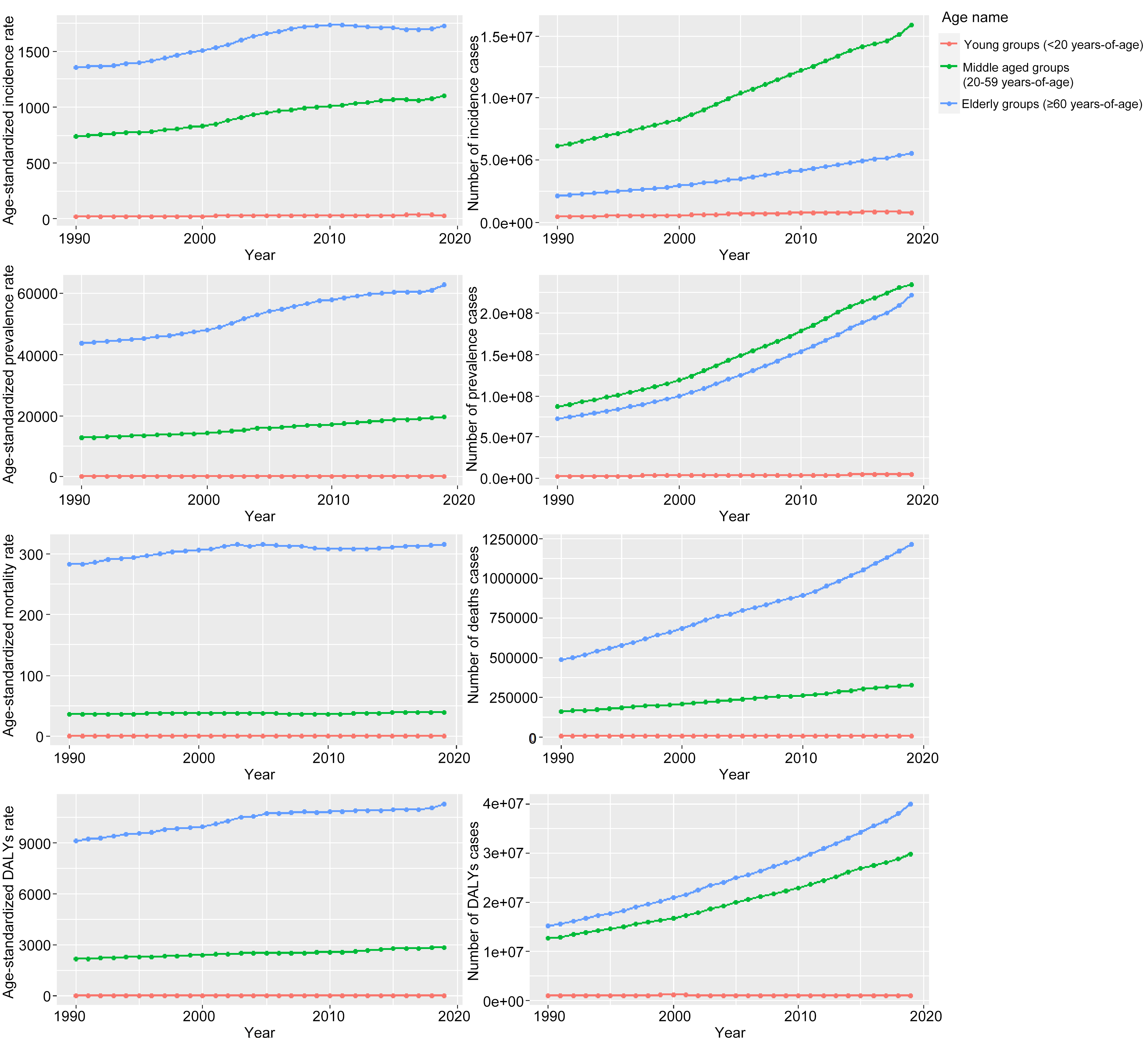
**

**Figure S6.** Trends in the disease burden of diabetes globally by age from 1990 to 2019

**
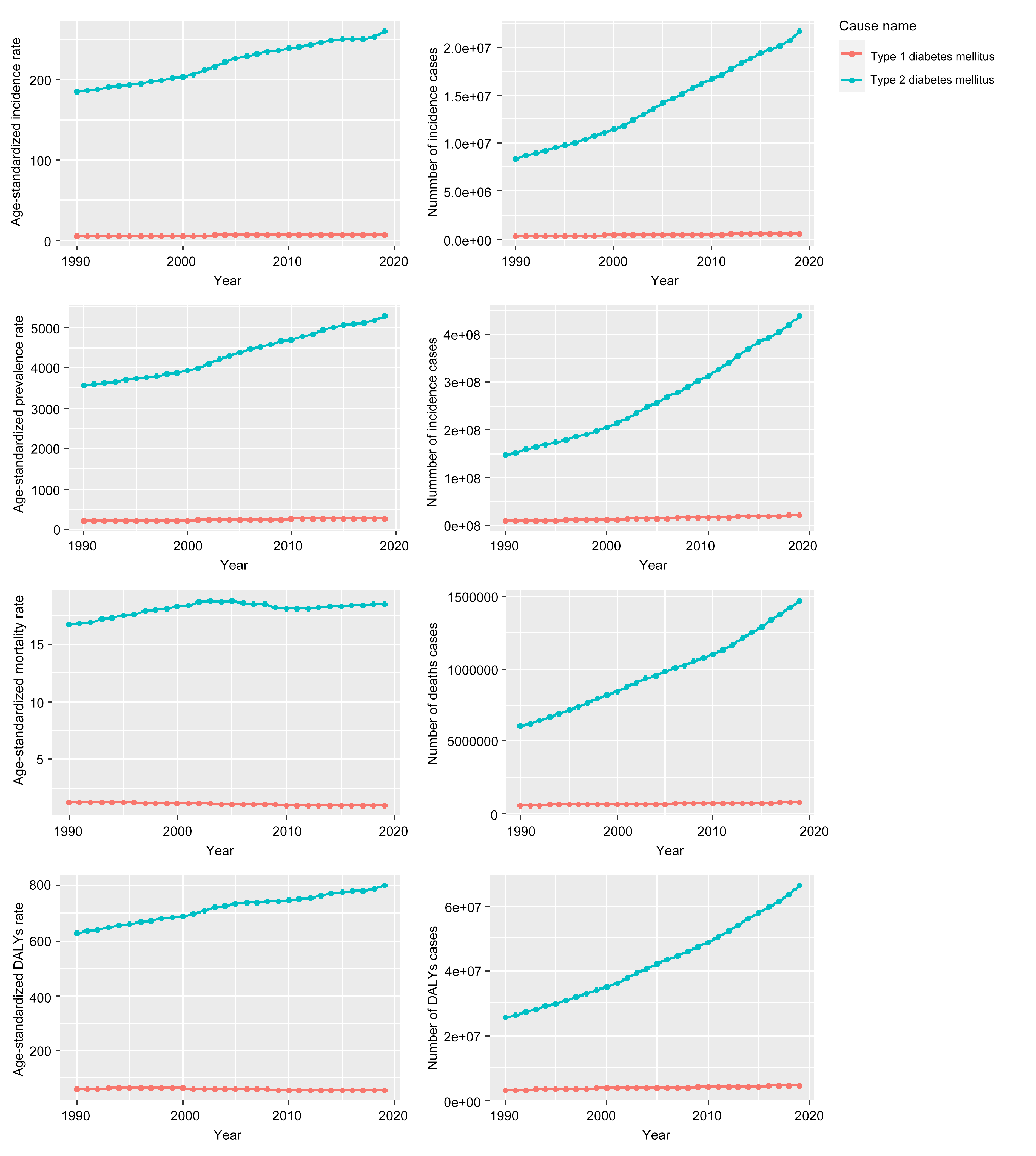
**

**Figure S7.** Trends in the disease burden across different subtypes of diabetes globally from 1990 to 2019

**
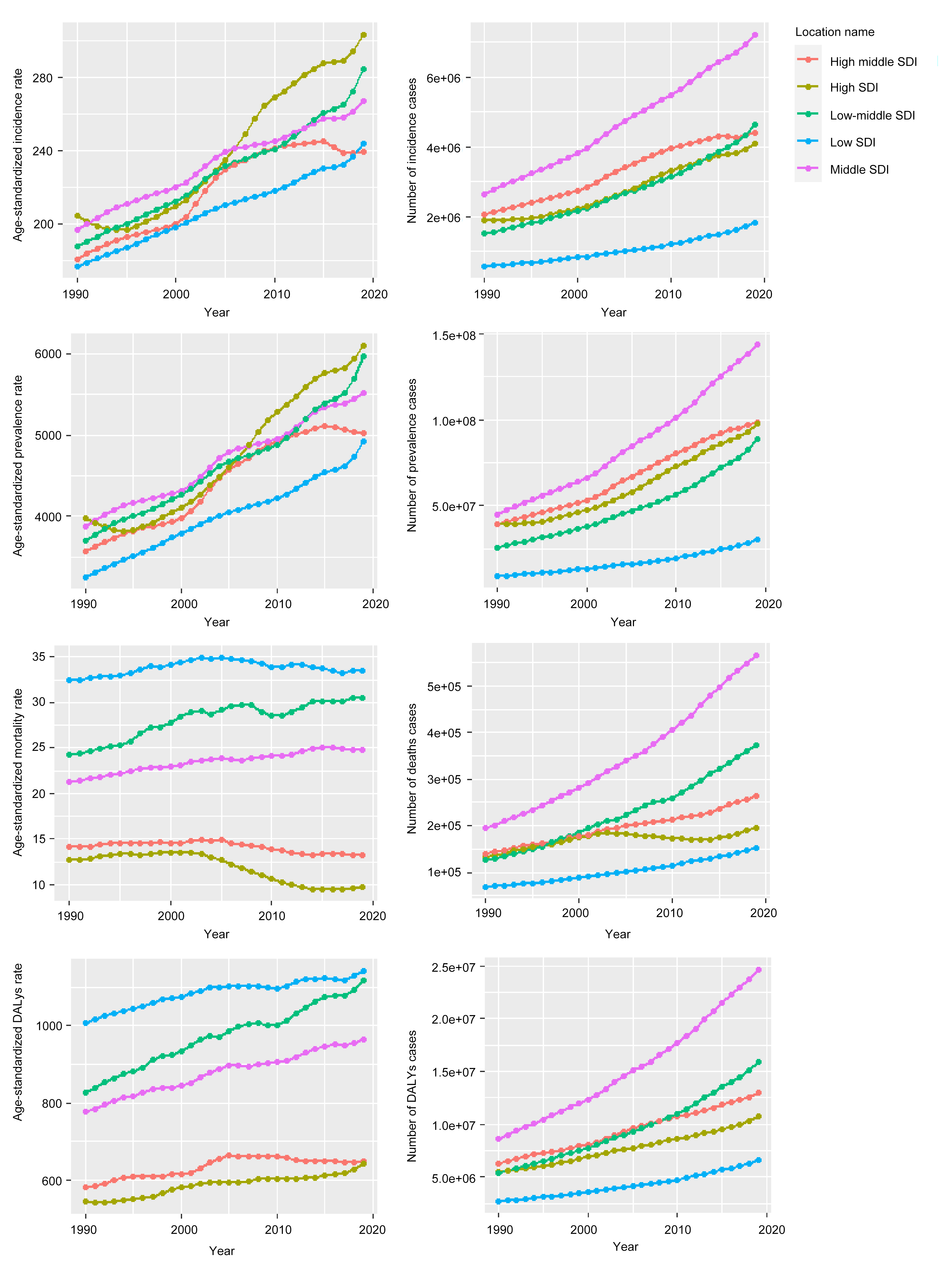
**

**Figure S8.** Trends in the disease burden of diabetes in different SDI regions globally from 1990 to 2019. **Abbreviation:** SDI: socio-demographic index


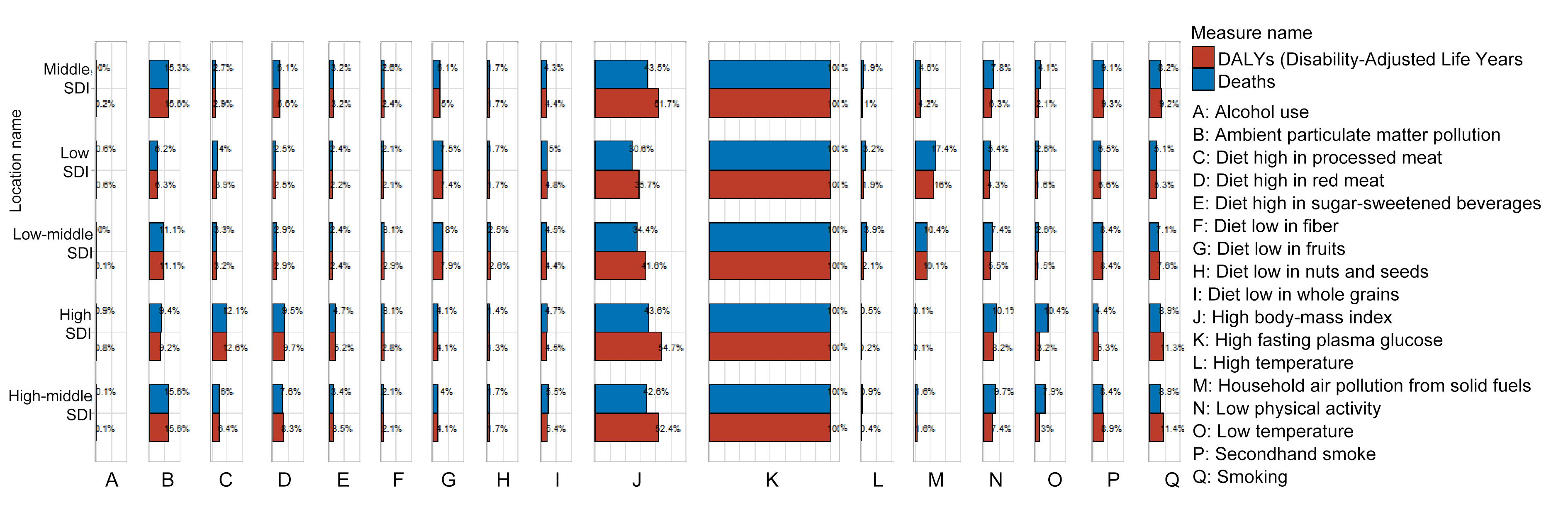


**Figure S9.** The relevant influencing factors associated with diabetes in all SDI regions. **Abbreviations:** SDI: socio-demographic index

**
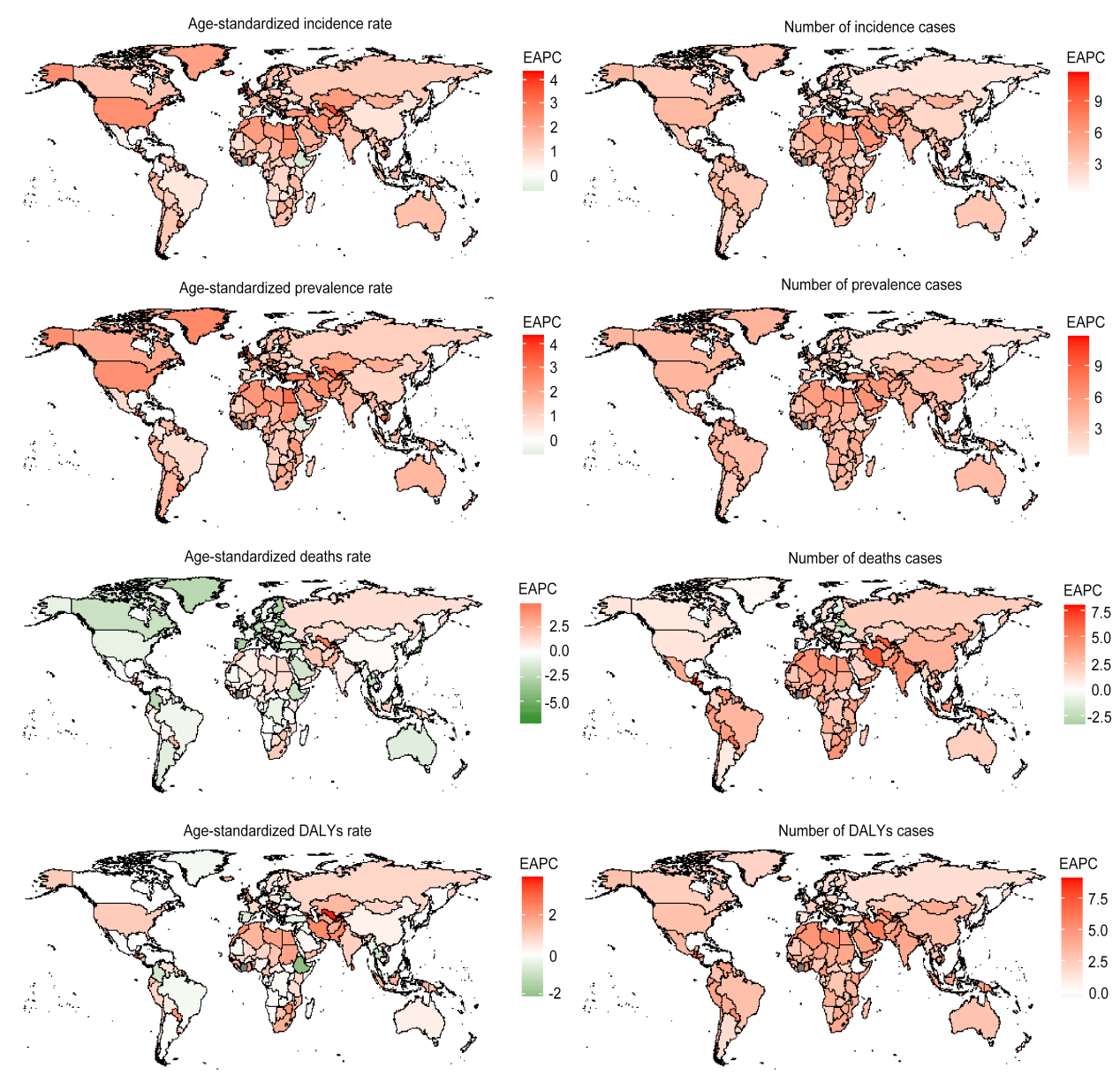
**

**Figure S10.** The EAPC value of diabetes ASR from 1990 to 2019. **Abbreviations:** EAPC: estimated annual percentage change; ASR: age-standardized rate


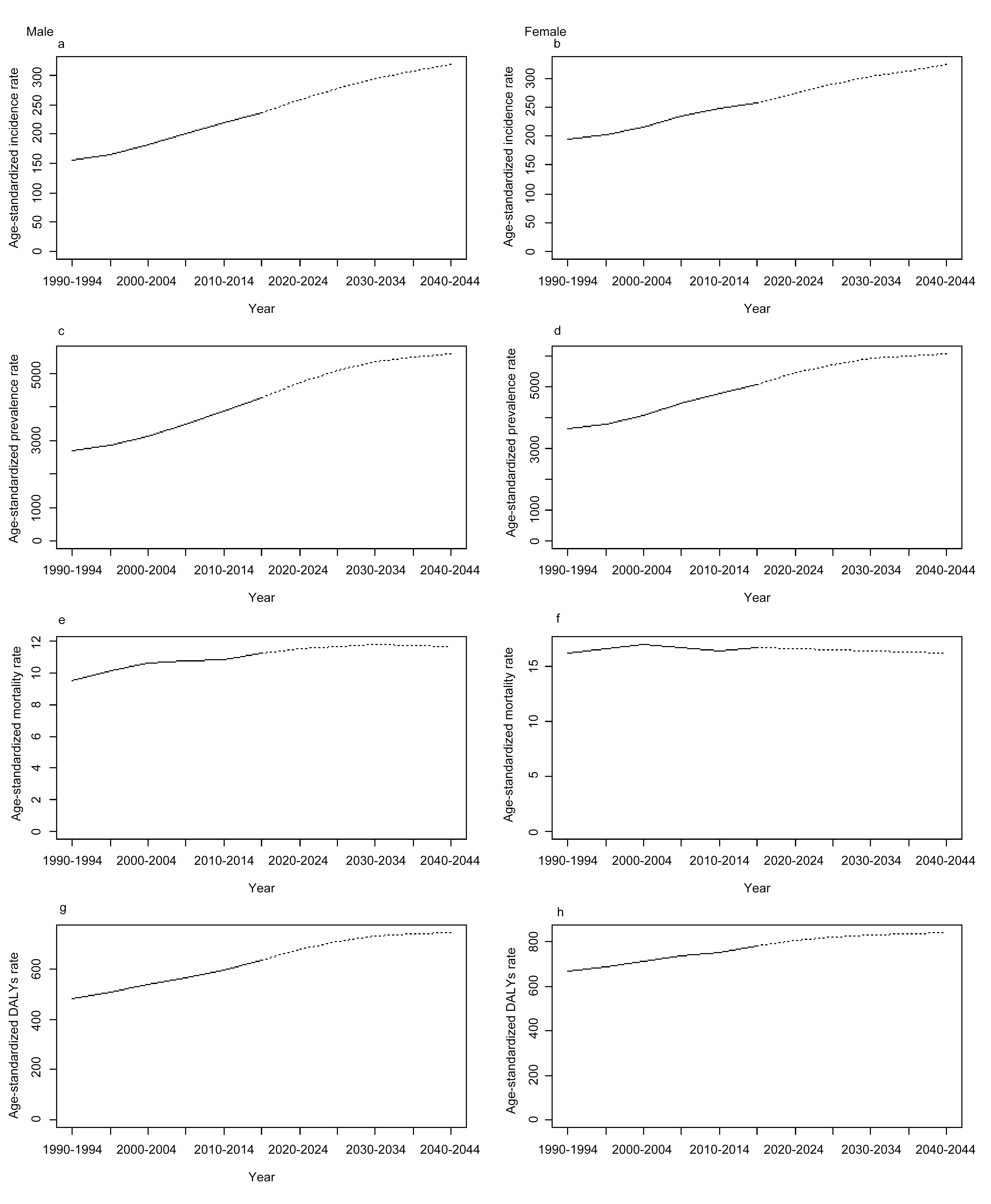


**Figure S11.** Trends in diabetes-related ASIR (a and b), the age-standardized prevalence rate (c and d), ASMR (e and f), and age-standardized DALYs rate (g and h) by sex globally from 1990 to 2044: observed (solid lines) and predicted rates of the APC model (dashed lines). **Abbreviations:** ASIR, age-standardized incidence rate; ASMR, age-standardized mortality rate; DALY: disability-adjusted life years; APC: age-period-cohort


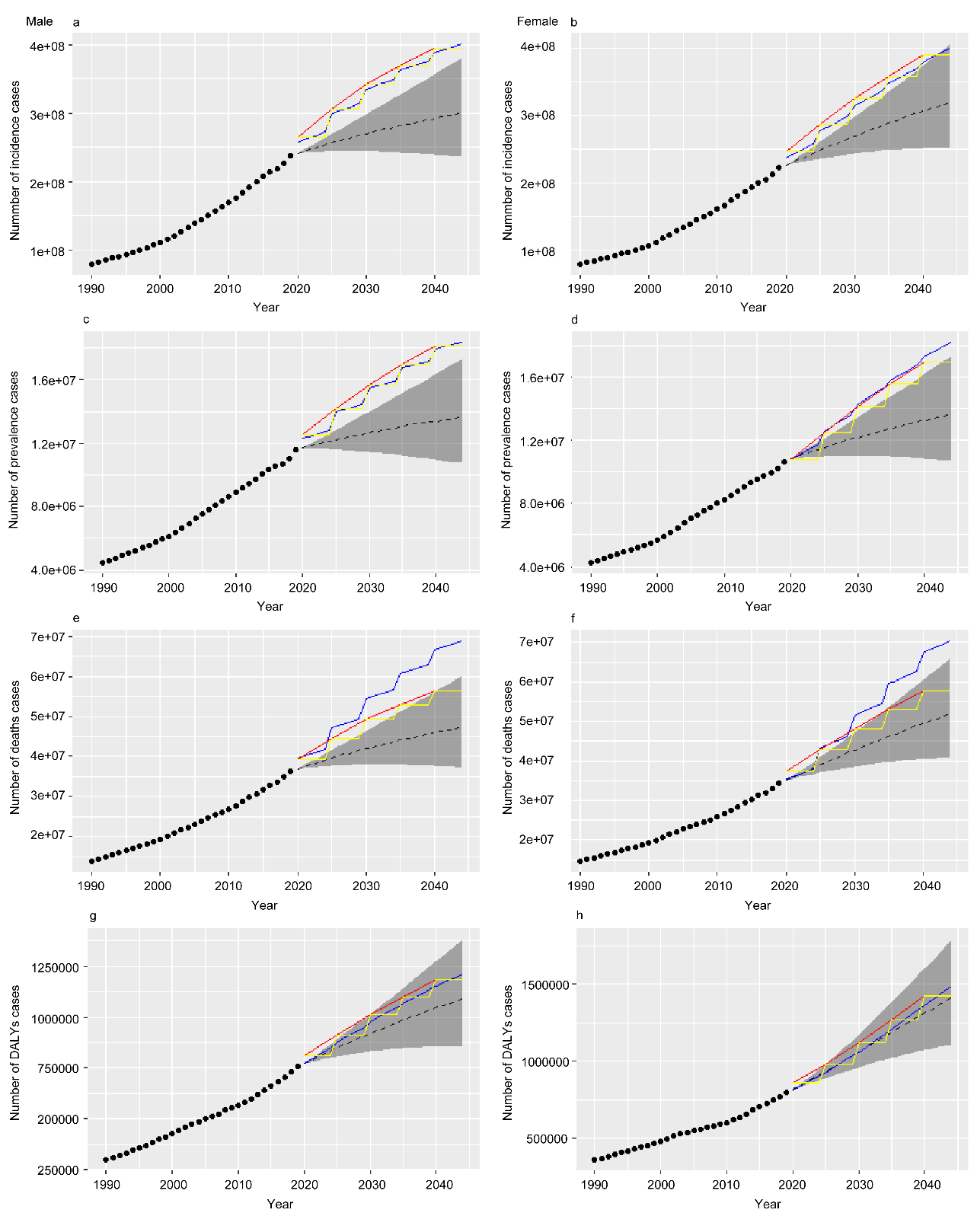


**Figure S12.** Trends in the number of incidence cases (a and b), the number of prevalence cases (c and d), number of deaths cases (e and f), and number of DALYs cases (g and h) attributable to diabetes by sex globally from 1990 to 2044: observed (before 2019) and predicted (after 2019) numbers of the APC model. Shading indicates if the rate remained stable (baseline reference), decreased by 1% per year (optimistic reference, lower limit), or increased by 1% per year (pessimistic reference, upper limit) based on the observed rate in 2019. Three methods were used in the prediction. The red line is calculated by the predicted rate of each 5-year group and average population size of the 5-year groups. The blue line method was used to calculate the rate of each group in terms of the predicted rate of each 5-year group and average population situation of the 5-year groups. The yellow line was calculated by the predicted rate of each 5-year group and annual population situation. **Abbreviations:** DALY: disability-adjusted life years; APC: age-period-cohort


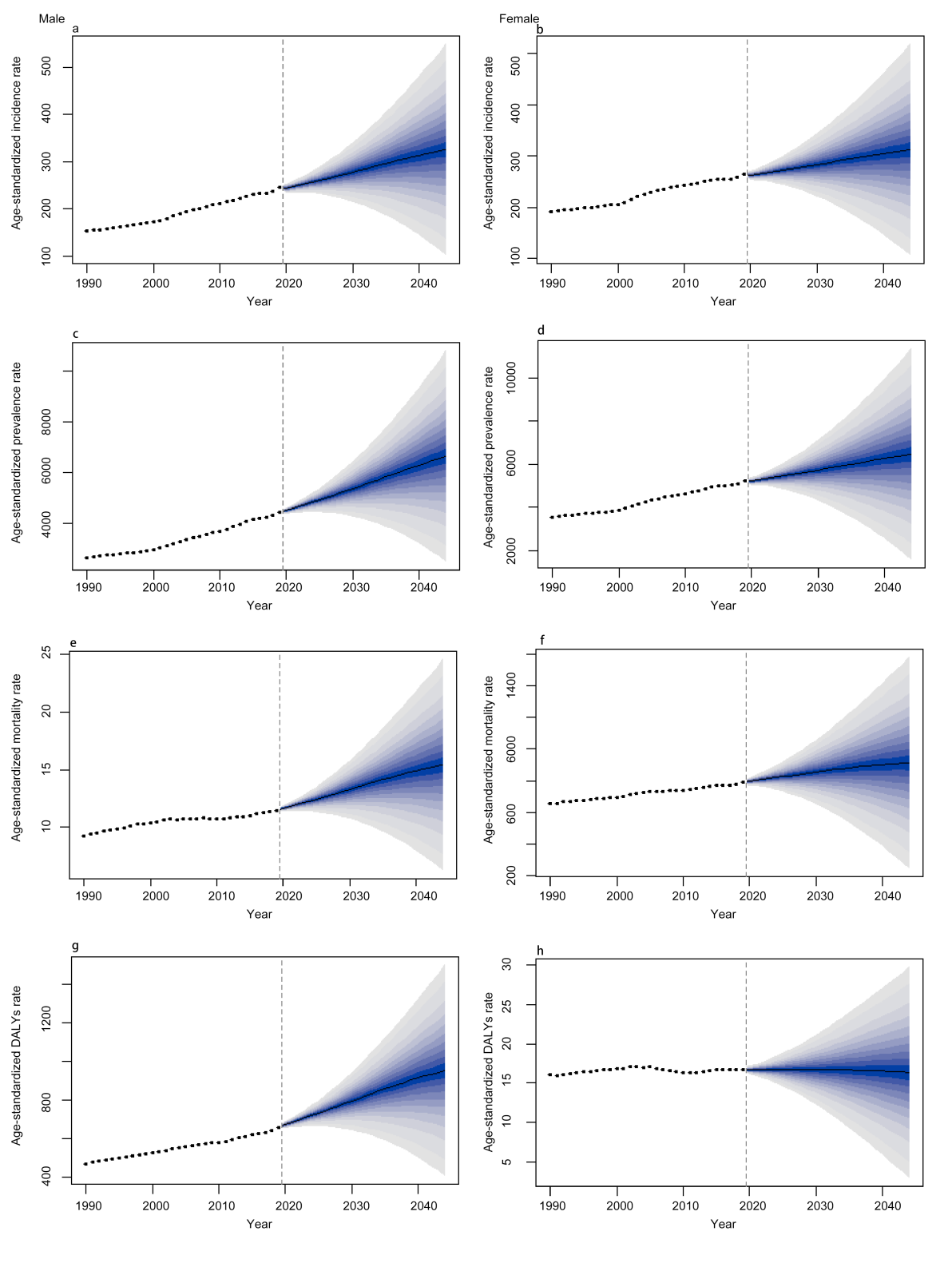


**Figure S13.** Trends in diabetes-related ASIR (a and b), the age-standardized prevalence rate (c and d), ASMR (e and f), and age-standardized DALYs rate (g and h) by sex globally from 1990 to 2044: observed (dashed lines) and predicted rates of the BAPC model (solid lines). The blue region shows the upper and lower limits of the 95% UIs. **Abbreviations:** ASIR, age-standardized incidence rate; ASMR, age-standardized mortality rate; DALY, disability-adjusted-life-year; BAPC: Bayesian age-period-cohort; UIs: uncertainty intervals


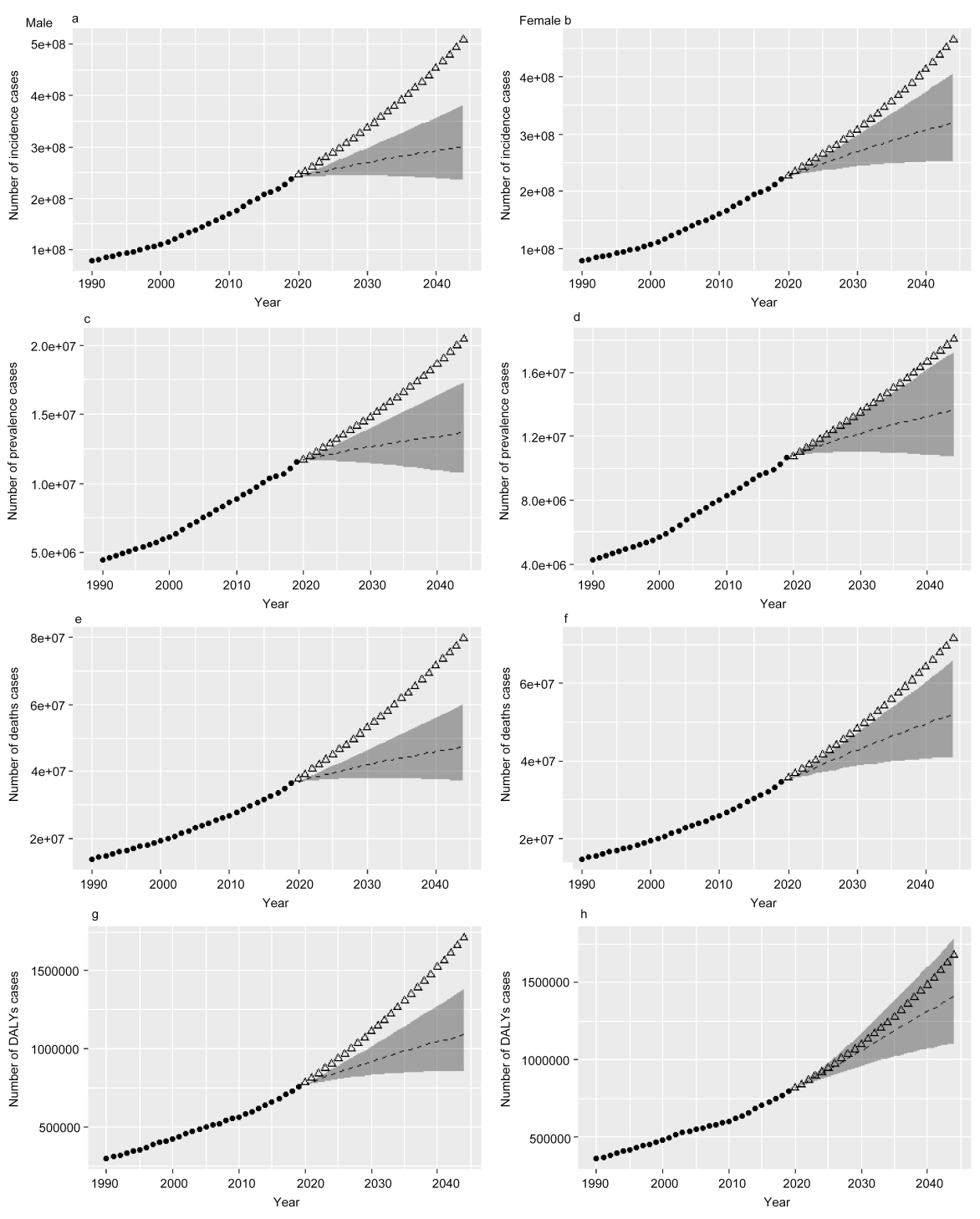


**Figure S14.** Trends in the number of incidence cases (a and b), the number of prevalence cases (c and d), number of deaths cases (e and f), and number of DALYs cases (g and h) attributable to diabetes by sex globally from 1990 to 2044: observed (before 2019) and predicted (after 2019) numbers provided by the BAPC model. Shading indicates if the rate remained stable (baseline reference), decreased by 1% per year (optimistic reference, lower limit), or increased by 1% per year (pessimistic reference, upper limit) based on the observed rate in 2019. The curve formed by the triangle is the prediction result of the BAPC model. **Abbreviations:** DALY, disability-adjusted-life-year; BAPC: Bayesian age-period-cohort


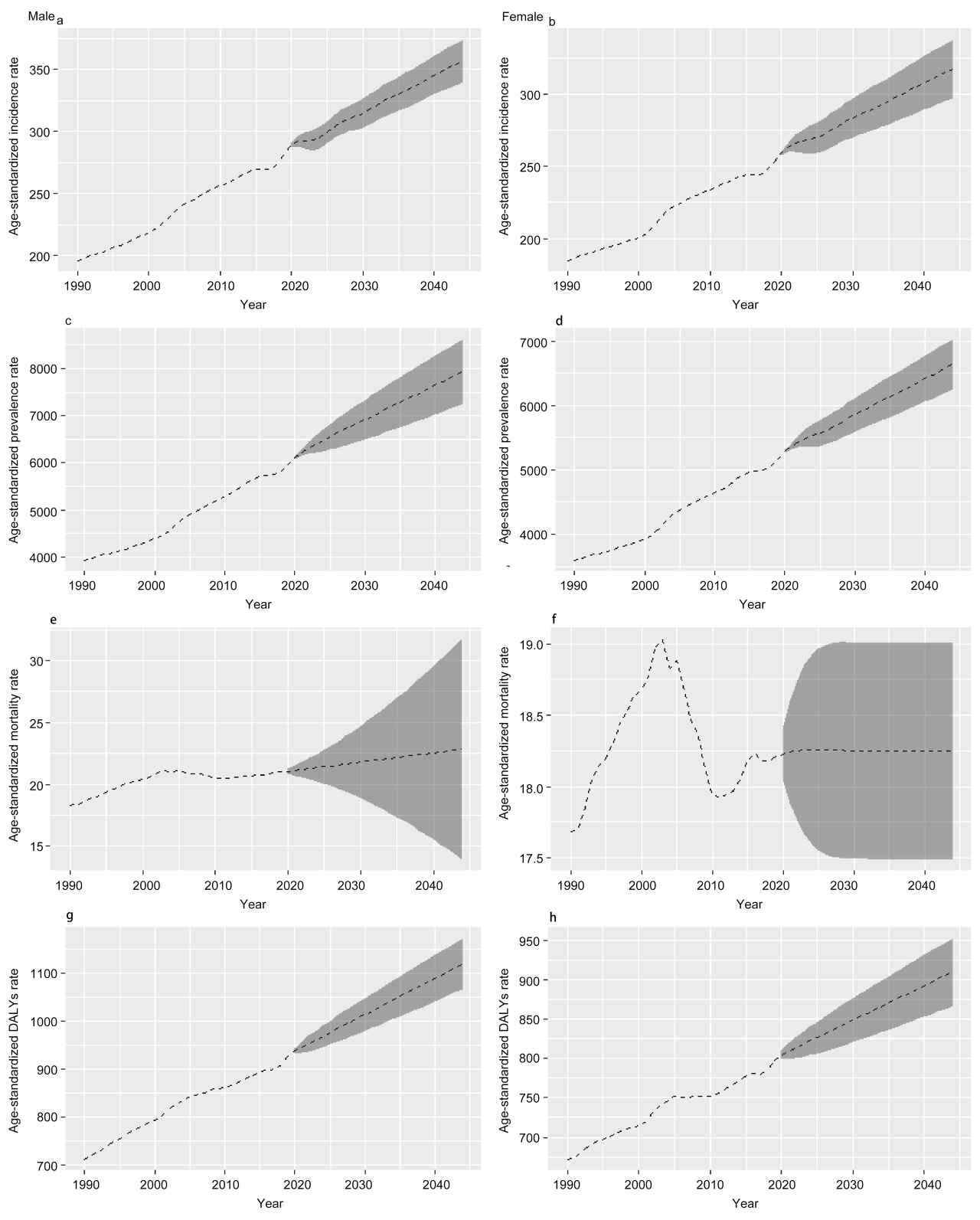


**Figure S15.** Trends in diabetes-related ASIR (a and b), the age-standardized prevalence rate (c and d), ASMR (e and f), and age-standardized DALYs rate (g and h) by sex globally from 1990 to 2044: observed (before 2019) and predicted rates of the ARIMA model (after 2019). Shading indicates the upper and lower limits of the 95% CIs. **Abbreviations:** ASIR, age-standardized incidence rate; ASMR, age-standardized mortality rate; DALY, disability-adjusted-life-years; ARIMA, autoregressive integrated moving average; CIs, confidence intervals


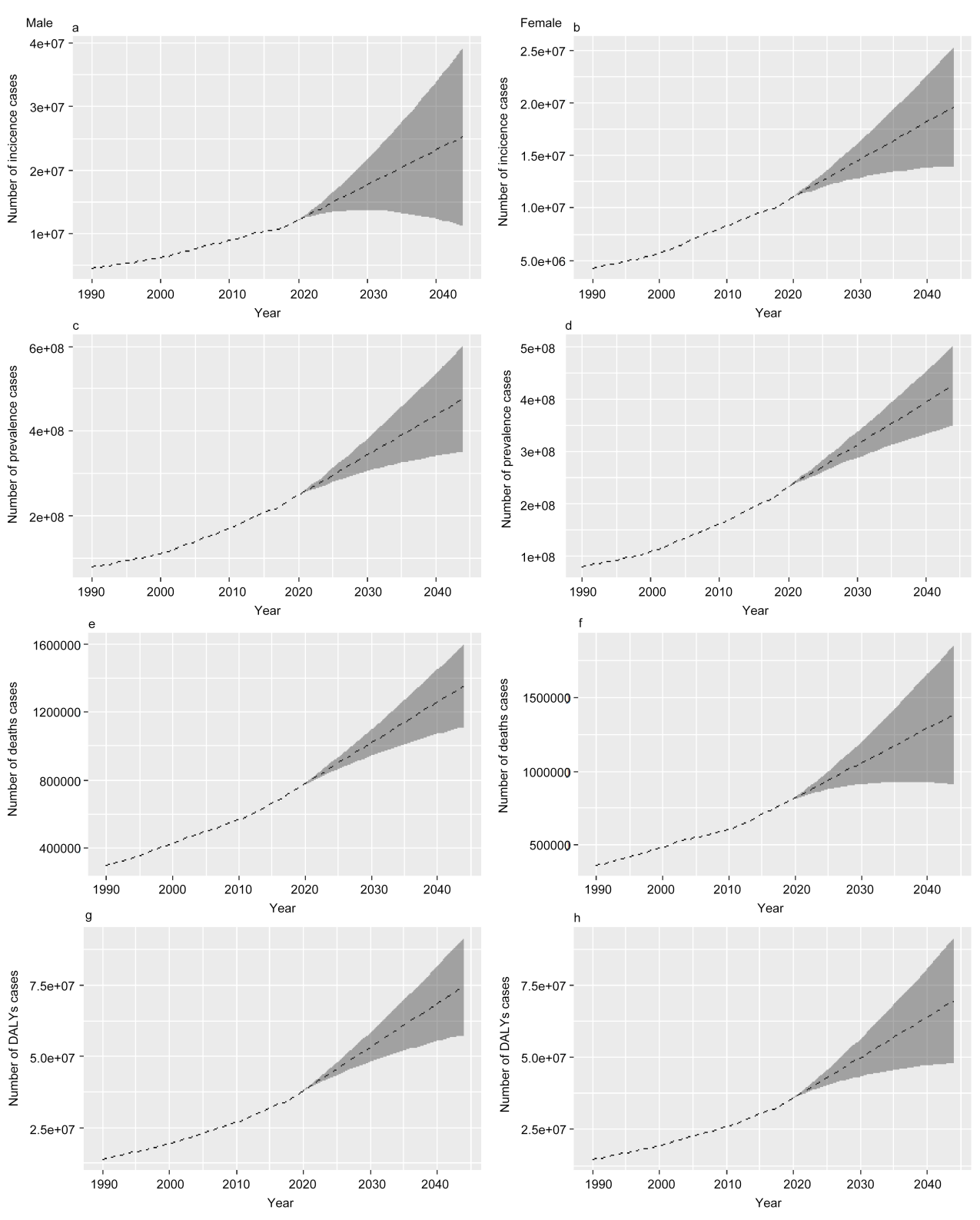


**Figure S16.** Trends in the number of incidence cases (a and b), the number of prevalence cases (c and d), number of death cases (e and f), and number of DALYs cases (g and h) attributable to diabetes by sex globally from 1990 to 2044: observed (before 2019) and predicted numbers of the ARIMA model (after 2019). Shading indicates the upper and lower limits of the 95% CIs. **Abbreviations:** DALY, disability-adjusted-life-years; ARIMA, autoregressive integrated moving average; CIs, confidence intervals


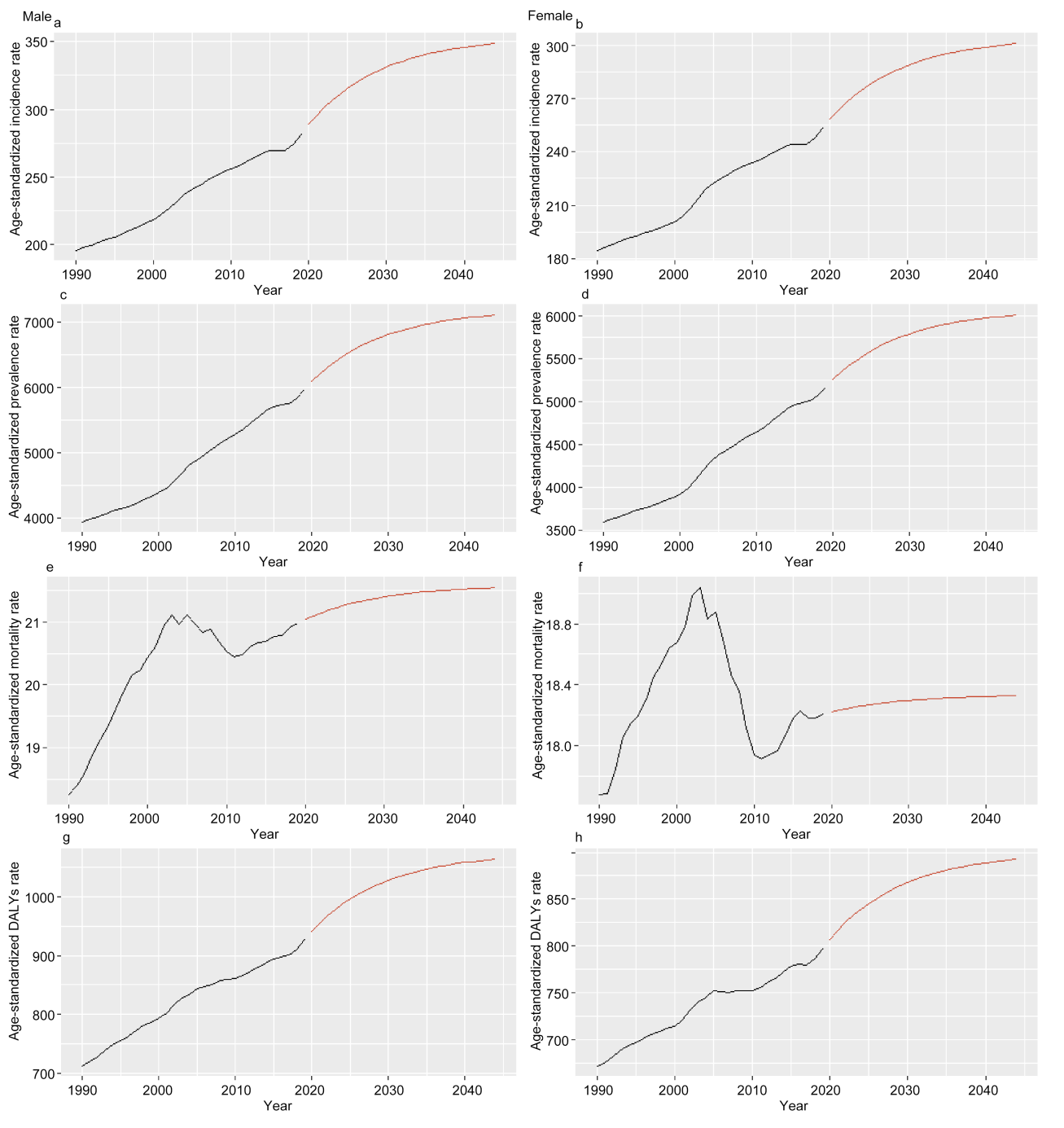


**Figure S17.** Trends in diabetes-related ASIR (a and b), the age-standardized prevalence rate (c and d), ASMR (e and f), and age-standardized DALYs rate (g and h) by sex globally from 1990 to 2044: observed (before 2019) and predicted rates of the ES model (after 2019). Shading indicates the upper and lower limits of the 95% CIs. **Abbreviations:** ASIR, age-standardized incidence rate; ASMR, age-standardized mortality rate; DALY, disability-adjusted-life-years; ES, exponential smoothing; CIs, confidence intervals


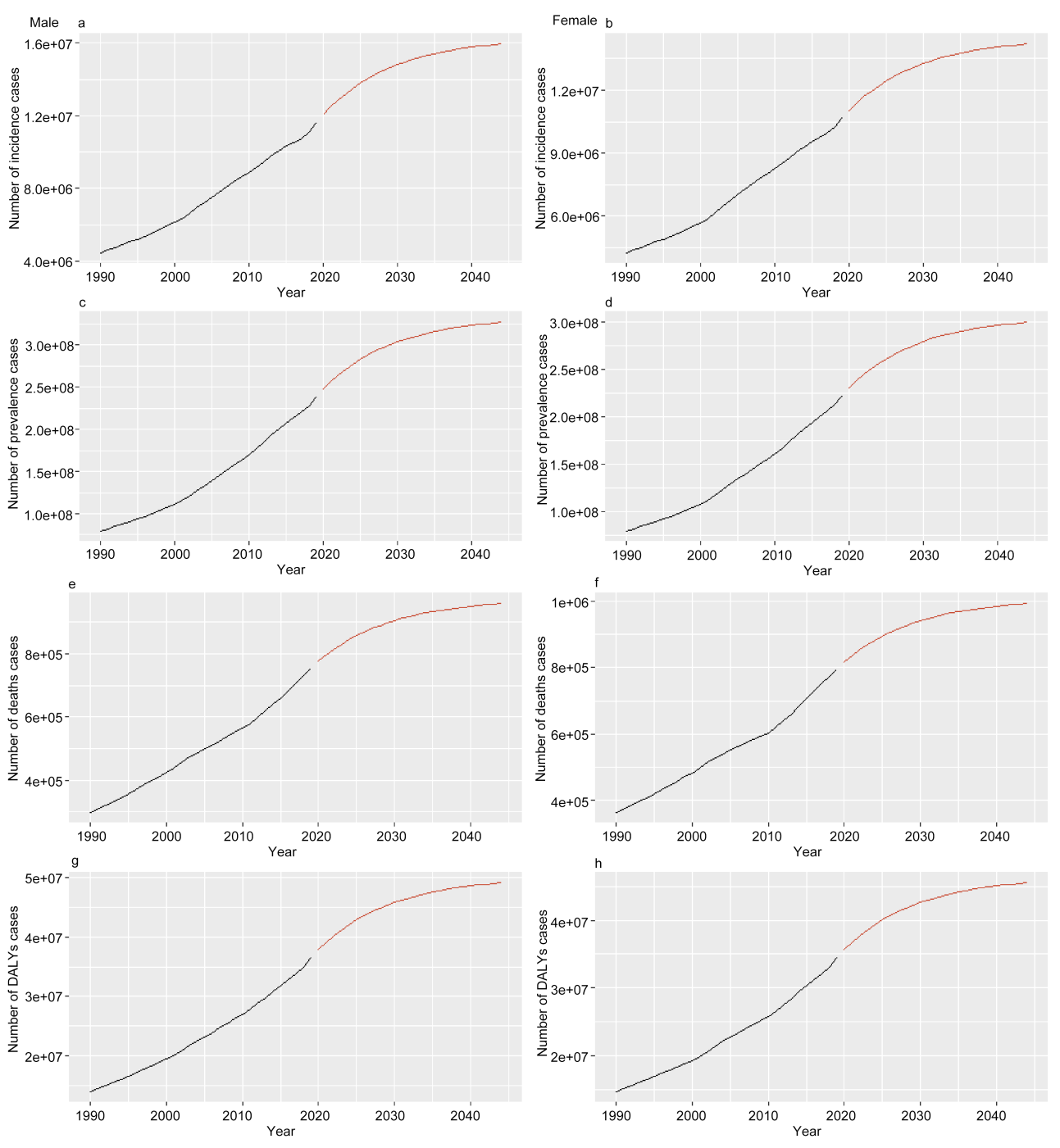


**Figure S18.** Trends in the number of incidence cases (a and b), the number of prevalence cases (c and d), number of death cases (e and f), and number of DALYs cases (g and h) attributable to diabetes by sex globally from 1990 to 2044: observed (before 2019) and predicted numbers provided by the ES model (after 2019). Shading indicates the upper and lower limits of the 95% CIs. **Abbreviations:** DALY, disability-adjusted-life-years; ES, exponential smoothing; CIs, confidence intervals
